# Different hypertension thresholds and cognitive decline: a pooled analysis of three ageing cohorts

**DOI:** 10.1186/s12916-021-02165-4

**Published:** 2021-11-01

**Authors:** Yanjun Ma, Rong Hua, Zhenchun Yang, Baoliang Zhong, Li Yan, Wuxiang Xie

**Affiliations:** 1 Peking University Clinical Research Institute, Peking University First Hospital, Beijing, China; 2 PUCRI Heart and Vascular Health Research Center at Peking University Shougang Hospital, Beijing, China; 3 Key Laboratory of Molecular Cardiovascular Sciences (Peking University), Ministry of Education, Beijing, China; 4 Department of Epidemiology and Biostatistics, School of Public Health, Imperial College London, London, UK; 5 Department of Geriatric Psychiatry, Affiliated Wuhan Mental Health Center, Tongji Medical College of Huazhong University of Science & Technology, Wuhan, China; 6 National School of Development, Peking University, Beijing, China

**Keywords:** Blood pressure, Hypertension, Cognitive dysfunction

## Abstract

**Background:**

The 2017 American College of Cardiology (ACC)/American Heart Association (AHA) guidelines for high blood pressure (BP) in adults came up with a new definition of hypertension with a threshold BP level of 130/80 mmHg. But the 2018 European Society of Cardiology (ESC)/European Society of Hypertension (ESH) guidelines adhered to a conventional hypertension definition as BP ≥ 140/90 mmHg. We aimed to compare the trajectories of cognitive decline between participants with BP < 130/80 mmHg in all BP measurement waves and others with all BP < 140/90 mmHg.

**Methods:**

This pooled analysis involved middle-aged and older participants from three nationally representative ageing cohorts, including the Health and Retirement Study (HRS), the English Longitudinal Study of Ageing (ELSA), and the China Health Retirement Longitudinal Study (CHARLS). Participants were divided into the Normal (BP < 130/80 mmHg on all occasions throughout the study), the Borderline (BP < 140/90 mmHg on all occasions throughout the study but not in the Normal group), and the High (the rest of participants) BP groups. Global cognitive *Z* score was calculated from tests on memory, executive function, and orientation.

**Results:**

A total of 17,590 participants (HRS 6964, median follow-ups 12 years; ELSA 5334, median follow-ups 16 years; CHARLS 5292, median follow-ups 7 years) were included. No significant difference in global cognitive decline rate was detected between the Normal and the borderline groups (men, pooled *β* = − 0.006 standard deviation [SD]/year; 95% confidence interval [CI], − 0.020 to 0.008; *P* = 0.377; women, pooled *β* = 0.006 SD/year; 95% CI − 0.005 to 0.018; *P* = 0.269). Participants in the High group had a significantly faster cognitive decline (men, pooled *β* = − 0.011 SD/year; 95% CI − 0.020 to − 0.002; *P* = 0.013; women, pooled *β* = − 0.017 SD/year; 95% CI − 0.026 to − 0.008; *P* < 0.001) than that in the Borderline group.

**Conclusions:**

Individuals in the Borderline group did not experience significantly faster cognitive decline compared with those in the Normal group. It might not be necessary for individuals with borderline BP (between 130/80 and 140/90 mmHg) to initiate antihypertension therapy in consideration of cognitive decline.

**Supplementary Information:**

The online version contains supplementary material available at 10.1186/s12916-021-02165-4.

## Background

The 2017 American College of Cardiology (ACC)/American Heart Association (AHA) guidelines for high blood pressure (BP) in adults came up with a new definition of hypertension with a threshold BP level of 130/80 mmHg [1]. But the 2018 European Society of Cardiology (ESC)/European Society of Hypertension (ESH) guidelines adhered to a conventional hypertension definition as BP ≥ 140/90 mmHg [2]. This difference leads to distinct strategies for individuals with a BP between the two thresholds and might impact on primary prevention of a large group of hypertension-related diseases, including dementia [1, 2]. As one of the most severe disorders in later life, dementia affected 50 million people and imposed a financial burden of US$1 trillion globally in 2018 [3]. Since people live longer, both the above figures are still increasing drastically [3]. Dementia cannot be cured but can be prevented or delayed during its long period of the preclinical phase [4].

Large epidemiological studies have demonstrated that midlife hypertension was significantly associated with an increased rate of cognitive decline and higher dementia risk in later life [5, 6]. Midlife hypertension, defined as BP ≥ 140/90 mmHg, was related to 1.55-fold excess global cognitive decline risk and 1.20-fold elevated dementia risk according to a systematic review of prospective studies [6]. BP lowering has been proved to reduce the risk of cognitive impairment or dementia among patients with hypertension [1, 2]. But most of the previous studies on the association between hypertension and cognitive decline were based on the conventional hypertension definition as BP ≥ 140/90 mmHg [1, 2, 6, 7]. The association of borderline hypertension, with BP between 130/80 and 140/90 mmHg, and cognitive decline remains not elucidated. What is more, the role of long-term BP in cognitive decline was rarely investigated. Herein, we sought to compare the trajectories of cognitive decline between two groups: (1) the Normal BP group included participants with BP < 130/80 mmHg in all BP measurement waves; (2) the Borderline BP group included participants with all BP < 140/90 mmHg, except those in the Normal BP group. Our hypothesis was that individuals in the Normal BP group would experience slower cognitive decline than those in the Borderline BP group.

## Methods

### Study population

The present study implemented a cross-sectional design using open-access data from three nationally representative longitudinal ageing cohorts: waves 8 to 14 (2006 to 2018) of the Health and Retirement Study (HRS), waves 0 to 9 (1998/1999/2001 to 2018/2019) of the English Longitudinal Study of Ageing (ELSA), and waves 1 to 4 (2011 to 2018) of the China Health Retirement Longitudinal Study (CHARLS), which shared overall design and a large group of variables including BP and cognitive scores [8–13]. These three cohorts were conformed to the Declaration of Helsinki and approved by the Health Sciences and Behavioral Sciences Institutional Review Board at the University of Michigan, the London Multicentre Research Ethics Committee, and the Peking University Institutional Review Board, respectively. The HRS randomly enrolled individuals over age 50 in the USA via a multistage area probability sampling design since 199 2[14]. The ELSA sample was from the Health Survey for England (HSE), which randomly recruited individuals aged 50 years or older living in England using postcode [10, 14]. The CHARLS randomly selected Chinese residents aged 45 years or older via multistage probability sampling since 2011 [12]. All participants gave written informed consent and followed at 2-year, occasionally 3-year, intervals.

Wave 8 (2006) in the HRS, wave 1 (2002/2003) in the ELSA, and wave 1 (2011) in the CHARLS were regarded as the baseline in the present study. The inclusion criteria in the present analysis were attending wave 8 in the HRS, wave 1 in the ELSA, or wave 1 in the CHARLS. The exclusion criteria included: (1) missing complete cognitive scores, or (2) having a confirmed diagnosis of dementia/psychiatric disorders at baseline, or (3) missing important covariate (sex), or (4) only taking BP measurement on < 3 occasions, or (5) without follow-up cognitive scores.

### Blood pressure

BP measurement was performed for half of the participants in waves 8, 10, 12, and 14 and the other half in waves 9, 11, and 13 using Omron HEM-780 Monitor in the HRS; waves 0, 2, 4, 6, and 8 in the ELSA using Omron HEM-907 Monitor; and waves 1 to 4 in the CHARLS using Omron HEM-7200 Monitor. In the ELSA, BP at wave 0 was regarded as the baseline BP since no BP measurement was conducted at wave 1. Participants were asked to remain seated and quiet during the measurement. The interviewers placed the cuff on the participants’ left arm. Three measurements were conducted at 1-min intervals in a single wave, the mean of which was used. The Normal BP group included participants with BP < 130/80 mmHg in all BP measurement waves. Except those in the Normal BP group, other participants with all BP below 140/90 mmHg were in the Borderline BP group. The other participants were in the High BP group. In other words, participants in the Normal BP group had their BP persistently under the threshold of hypertension definition in the ACC/AHA guidelines. Participants in the Borderline BP group failed to keep their BP persistently under the ACC/AHA threshold, but successfully under the ESC/ESH threshold. The High BP group contained participants with BP reaching the ESC/ESH threshold on at least one occasion.

### Cognitive assessments

Cognitive assessments were conducted in waves 8 to 14 in the HRS, waves 1 to 9 in the ELSA, and waves 1 to 4 in the CHARLS. Three cognitive domains were covered, including memory, executive function, and orientation.

Immediate and delayed recall of ten unrelated words was used for the memory test. One point was given for each word recalled in either immediate or delayed recall task. Orientation was evaluated by four questions on the year, the month, the date of the month, and the day of the week. The sum of correct answers was regarded as the orientation score. The scores of memory and orientation ranged 0 to 20 and 0 to 4, respectively. Executive function was assessed by the counting-backward test (0 to 2 points) and the Serial Sevens test (0 to 5 points) in the HRS (0 to 7 points in total); an animal fluency test in the ELSA; an intersecting pentagon copying test (0 or 3 points); and the Serial Sevens test (0 to 5 points) in the CHARLS (0 to 8 points in total). In the counting-backward test, participants counted backwards from 20 as quickly as they could. A successful count from 19 to 10 or 20 to 11 on the first try was given two points; otherwise, success on the second try was given one point. In the Serial Sevens test, participants counted backwards from 100 by sevens for five times. One point was given for each correct answer. In the animal fluency test, the participant was asked to name animals as many as he or she could in 1 min. The score equalled the number of animals named, thus there was no upper limit of score range. In the intersecting pentagon copying test, participants were asked to observe and then draw a picture of two overlapping pentagons. A successful drawing was given three points. Both the validity and the reliability of these tests have been well documented [15–18]. Full details of these tests can be found in Additional file 1: Supplemental Methods.

To evaluate the global cognitive function, *Z* scores were generated by two steps in each cohort. Step 1: the domain *Z* scores were generated by standardizing to baseline. Each domain test score was subtracted by the mean and then divided by the standard deviation (SD) of the baseline domain scores. For instance, the mean and SD of executive function at baseline in the HRS were 5.84 and 1.35, respectively. Then the executive *Z* score at any wave was calculated as (original executive score at the wave − 5.84) / 1.35. Step 2: the global *Z* score of an individual at each wave was calculated from the mean score of three domains by re-standardizing to baseline. This procedure to generate cognitive *Z* scores has been widely accepted [15–20].

### Covariates

A large group of potential confounders for the association between BP and cognitive function were selected for our analyses, including sex, age (years), race, body mass index (BMI, kg/m^2^), education, cohabitation status, current smoking, alcohol consumption, exercise, depressive symptoms, antihypertension medication, diabetes, hypercholesterolemia, coronary heart disease, stroke, cancer, and chronic lung disease in the present analysis. Race was divided into white or not, thus race was not included in the analysis of the CHARLS. High level of education was defined as ≥ the senior level of high school or ≥ 12 years of education. Cohabitation status was categorized as living alone or not. Current smoking indicated smoking currently or not. Once per week or a higher frequency of drinking was defined as alcohol consumption. Engaging in vigorous or moderate activities once weekly or more was regarded as physically active. Depressive symptoms were evaluated by an 8-item version of the Center for Epidemiologic Studies Depression (CESD-8, one point for each item) in the HRS and the ELSA, and a 10-item version of the CESD (CESD-10, 0 to 3 points for each item) in the CHARLS. Participants with a score of ≥ 4 in the CESD-8 or ≥ 12 in the CESD-10 were regarded as having depressive symptoms [21, 22]. The proportion of waves reporting any antihypertension medication using in all attending waves was used. For example, if a participant attended four waves throughout the study and reported antihypertension medication using at two waves, then the proportion would be 0.5. Chronic disease measures included self-reported physician-diagnosed diabetes (or current use of anti-diabetic therapy), coronary heart disease, stroke, cancer, and chronic lung disease. Hypercholesterolemia was defined as self-reported physician-diagnosed hypercholesterolemia, or self-reported use of lipid-lowering medication, or total cholesterol ≥ 240 mg/dL [23]. Full details of covariates can be found in Additional file 1: Supplemental Methods.

### Statistical analysis

The results are presented as the mean ± SD or the median with the interquartile range (IQR) for continuous variables and numbers (percentage) for categorical variables. Association between BP group and global or domain cognitive *Z* score decline (SD/year) during follow-up in a single cohort was evaluated by linear mixed models including BP group, time (duration since baseline), BP group × time, age at baseline, and other covariates mentioned above. The Borderline BP group was regarded as the reference group. The cognitive decline rate of the Normal or the High BP group compared with that of the Borderline BP group was indicated by the regression coefficient of BP group × time. Linear mixed models can also handle missing data. Random-effect meta-analyses were performed for the pooled estimates. The percentage of variation across cohorts due to heterogeneity rather than chance was indicated by the *I*^2^ statistic in meta-analyses. The approach that pooling coefficients of linear mixed models has been used in previous studies [24–26].

Five sensitivity analyses were performed. The first excluded participants who ever reported antihypertension medication using either at baseline or during follow-up. The second excluded those with hypotension, defined as SBP < 90 mmHg or DBP < 60 mmHg, on at least one BP measurement occasion over the study. The third excluded individuals with stroke or coronary heart disease at baseline. The fourth included all participants who had complete baseline data, at least one reassessment of cognitive function, and BP measurements on ≥ 2 occasions. The last sensitivity analysis was performed using original cognitive scores rather *Z* scores. In addition, a non-response analysis was performed to compare the baseline characteristics between participants who were included and those who were excluded due to less than three BP measurements or no follow-up cognitive score.

All analyses were conducted by sex, using SAS (version 9.4; SAS Institute Inc) and STATA (version 11; Stata Corp LLC). All tests were two-sided with an alpha of 0.05 as the threshold for statistical significance.

## Results

### Sample size and baseline characteristics

Among 48,274 participants (HRS 18,469; ELSA 12,099; CHARLS 17,706) attending baseline surveys, 17,590 participants with complete baseline data, BP measurements on ≥ 3 occasions, and at least one reassessment of cognitive function were included in our analyses, including 6964 from the HRS (mean age 66.3 ± 8.0 years, women 59.6% [4154], median follow-up duration: 12.0 [IQR 10.0 to 12.0] years), 5334 from the ELSA (mean age 62.4 ± 8.9 years, women 56.2 % [2996], median follow-up duration 16 [IQR 12 to 16] years), and 5292 from the CHARLS (mean age 58.1 ± 8.8 years, women 52.1% [2759], median follow-up duration 7 [IQR 4 to 7] years). The number and proportion of participants completing planned BP measurement waves of each cohort are shown in Additional file 1: Table S1. BP groups, covariates, and baseline cognitive scores by domain are presented by sex in Table 1 and Additional file 1: Table S2. The three cohorts exhibited significant differences in baseline characteristics (Additional file 1: Table S2). The HRS participants were the oldest, had the highest proportion of those with high-level education, and had the highest prevalence of most chronic diseases (except chronic lung disease). The ELSA participants presented the highest systolic BP and had the highest proportion of the High BP group and alcohol consumption. The CHARLS participants were the youngest, exhibited the lowest body mass index and the highest memory cognitive score, but had the highest percentage of physical inactive, depressive symptoms, smoking, and chronic lung disease.

**Table 1:** BP group and covariates of participants in analyses

Characteristic	HRS (***n*** = 6964)			ELSA (***n*** = 5334)			CHARLS (***n*** = 5292)		
	Men (***n*** = 2810)	Women (***n*** = 4154)	***P*** value*	Men (***n*** = 2338)	Women (***n*** = 2996)	***P*** value*	Men (***n*** = 2533)	Women (***n*** = 2759)	***P*** value*
Age (years)	66.4 ± 7.9	66.2 ± 8.0	0.420	62.2 ± 8.7	62.5 ± 9.1	0.202	59.4 ± 8.6	56.9 ± 8.8	< 0.001
White (%)	2376 (84.6)	3369 (81.1)	< 0.001	2291 (98.0)	2946 (98.3)	0.355	0 (%)	0 (%)	
BP group (%)			< 0.001			< 0.001			< 0.001
Normal	378 (13.5)	736 (17.7)		186 (8.0)	395 (13.2)		598 (23.6)	820 (29.7)	
Borderline	700 (24.9)	1044 (25.1)		460 (19.7)	502 (16.8)		616 (24.3)	612 (22.2)	
High	1732 (61.6)	2374 (57.1)		1692 (72.4)	2099 (70.1)		1319 (52.1)	1327 (48.1)	
BMI (kg/m^2^)	29.4 ± 4.8	29.5 ± 6.0	0.551	27.5 ± 3.8	27.5 ± 5.0	0.814	23.1 ± 3.5	24.2 ± 3.8	< 0.001
Systolic blood pressure (mmHg)	133.4 ± 18.4	128.4 ± 19.7	< 0.001	141.9 ± 17.6	139.7 ± 20.2	< 0.001	129.9 ± 19.8	129.4 ± 21.4	0.313
Diastolic blood pressure (mmHg)	80.4 ± 11.1	79.4 ± 11.3	0.010	81.6 ± 11.3	74.8 ± 11.6	< 0.001	76.2 ± 12.2	75.5 ± 11.9	0.049
High level of education (%)	2326 (82.8)	3384 (81.5)	0.162	1043 (44.6)	832 (27.8)	< 0.001	387 (15.3)	193 (7.0)	< 0.001
Living alone (%)	457 (16.3)	1533 (36.9)	< 0.001	491 (21.0)	1101 (36.7)	< 0.001	206 (8.1)	326 (11.8)	< 0.001
Current smoking (%)	344 (12.2)	473 (11.4)	0.276	333 (14.2)	489 (16.3)	0.037	1866 (73.7)	203 (7.4)	< 0.001
Alcohol consumption (%)	1334 (47.5)	1233 (29.7)	< 0.001	1740 (74.4)	1595 (53.2)	< 0.001	1161 (45.8)	192 (7.0)	< 0.001
Physically active (%)	2399 (85.4)	3161 (76.1)	< 0.001	2027 (86.7)	2397 (80.0)	< 0.001	786 (31.0)	843 (30.6)	0.708
Depressive symptoms (%)	231 (8.2)	603 (14.5)	< 0.001	207 (8.9)	500 (16.7)	< 0.001	461 (18.2)	797 (28.9)	< 0.001
Taking antihypertension medication (%)	1982 (70.5)	2958 (71.2)	0.543	1203 (51.5)	1512 (50.5)	0.474	573 (25.6)	555 (23.6)	0.124
History of diseases									
Hypertension (%)	1729 (61.5)	2502 (60.2)	0.276	797 (34.1)	1055 (35.2)	0.392	986 (38.9)	1081 (39.2)	0.85
Diabetes (%)	522 (18.6)	671 (16.2)	0.008	165 (7.1)	129 (4.3)	< 0.001	122 (4.8)	169 (6.1)	0.037
Hypercholesterolemia (%)	1343 (47.8)	1656 (39.9)	< 0.001	829 (35.5)	1132 (37.8)	0.08	384 (15.2)	463 (16.8)	0.108
Heart disease (%)	682 (24.3)	707 (17.0)	< 0.001	433 (18.5)	441 (14.7)	< 0.001	235 (9.3)	344 (12.5)	< 0.001
Stroke (%)	128 (4.6)	133 (3.2)	0.004	63 (2.7)	75 (2.5)	0.662	47 (1.9)	38 (1.4)	0.167
Cancer (%)	386 (13.7)	472 (11.4)	0.003	92 (3.9)	202 (6.7)	< 0.001	17 (0.7)	28 (1.0)	0.174
Chronic lung disease (%)	177 (6.3)	341 (8.2)	0.003	103 (4.4)	154 (5.1)	0.214	278 (11.0)	232 (8.4)	0.002
Cognitive scores									
Memory	10.0 ± 3.1	10.9 ± 3.2	< 0.001	10.1 ± 3.2	10.5 ± 3.2	< 0.001	15.2 ± 4.7	15.0 ± 4.9	0.071
Executive^†^	7 (5–7)	6 (5–7)	< 0.001	21.1 ± 6.3	20.3 ± 6.0	< 0.001	7 (5–8)	5 (3–8)	< 0.001
Orientation	4 (4–4)	4 (4–4)	< 0.001	4 (4–4)	4 (4–4)	0.003	4 (3–4)	3 (2–4)	< 0.001

Data are presented as mean ± SD, *n* (%), or median (IQR)

* *P* value for differences between men and women

^†^ The executive scores are presented in different forms due to different assessment methods

*HRS*, the Health and Retirement Study; *ELSA*, the English Longitudinal Study of Ageing; *CHARLS*, the China Health Retirement Longitudinal Study; *BP*, blood pressure; *BMI*, body mass index

### BP group and cognitive decline

**Fig. 1:**
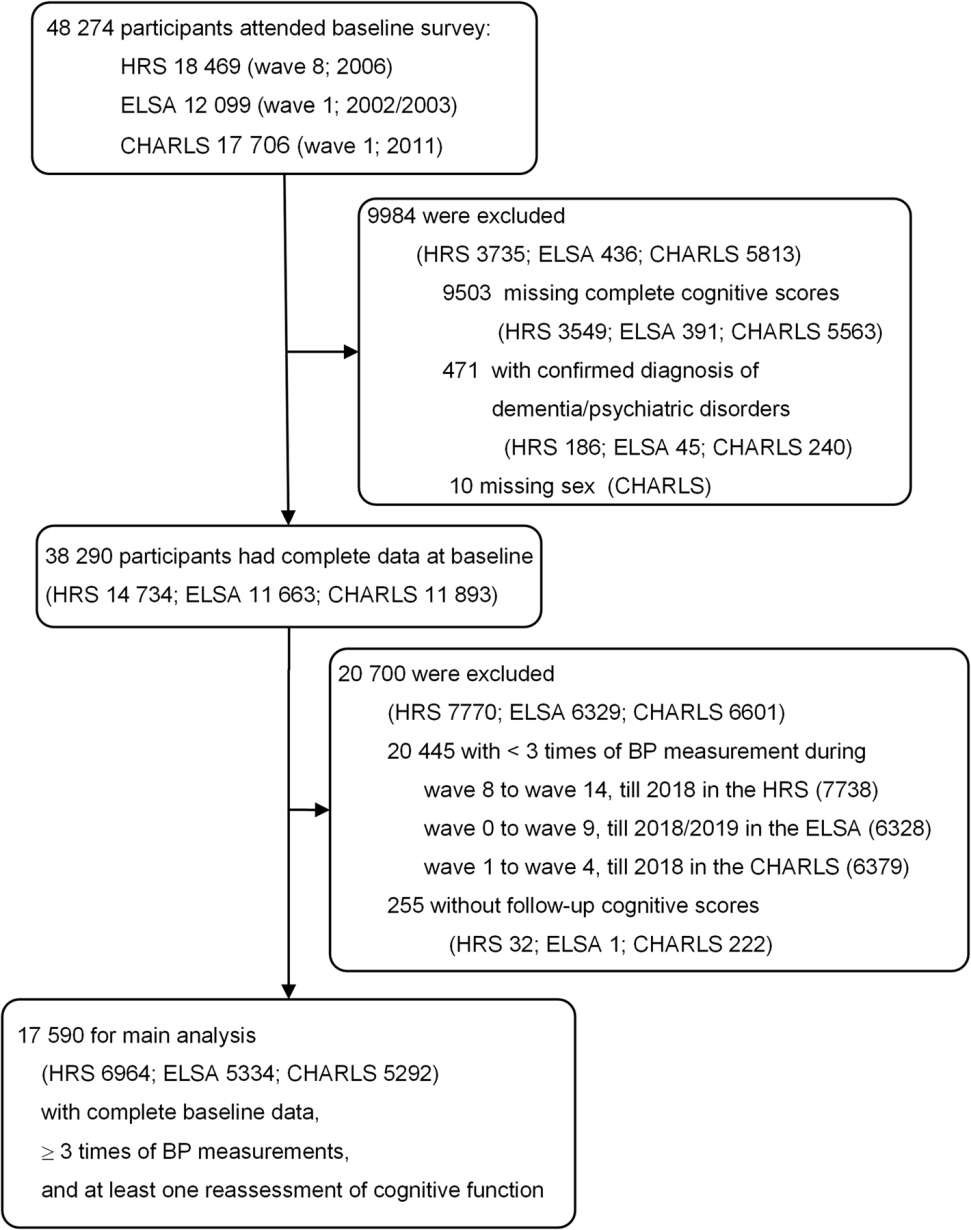
Flow chart of participant selection for this study

Due to the considerable (*I*^2^ over 75% according to Cochrane Handbook) between-cohort heterogeneity in the pooled analysis of global cognitive decline between the Normal and the Borderline BP groups, subsequent analyses were performed by sex (Additional file 1: Fig. S1 and Table S3) [27]. After adjustment for covariates, no significant difference was detected for global cognitive decline rate between the Normal and the Borderline BP groups (men, pooled *β* = − 0.006 SD/year; 95% confidence interval [CI], − 0.020 to 0.008; *P* = 0.377; women, pooled *β* = 0.006 SD/year; 95% CI − 0.005 to 0.018; *P* = 0.269; Fig. 1 and Tables 2 and 3). The High BP group presented a significantly faster global cognitive decline than the Borderline BP group (men, pooled *β* = − 0.011 SD/year; 95% CI − 0.020 to − 0.002; *P* = 0.013; women, pooled *β* = − 0.017 SD/year; 95% CI − 0.026 to − 0.008; *P* < 0.001; Fig. 1 and Tables 2 and 3). Similar trends were observed in analyses for single cognitive domains, except memory and orientation in men and executive function in women (where the difference between the High and the Borderline BP groups was not significant; Fig. 1 and Tables 2 and 3). Compared with individuals in the Normal BP group, those in the High BP experienced a significantly faster global cognitive decline in women (pooled *β* = − 0.023 SD/year; 95% CI − 0.039 to − 0.006; *P* = 0.007), while no significant difference was detected in men (pooled *β* = − 0.005 SD/year; 95% CI − 0.015 to 0.005; *P* = 0.326; Additional file 1: Fig. S2 [part A] and Table S4).

**Table 2:** Mean differences in rate of cognitive change (SD/year) during follow-up in men

BP Group	HRS (***n*** = 2810)		ELSA (***n*** = 2338)		CHARLS (***n*** = 2533)		Pooled analysis (***n*** = 7681)			
	***β*** (95% Cl)*	***P*** value	***β*** (95% Cl)*	***P*** value	***β*** (95% Cl)*	***P*** value	Pooled ***β*** (95% Cl)*	***P*** value	***I***^**2**^ (%)	***P*** value
Global										
Normal	−0.018 (− 0.032 to − 0.005)	0.008	0.005 (−0.012 to 0.023)	0.551	−0.003 (− 0.020 to 0.013)	0.687	− 0.006 (− 0.020 to 0.008)	0.377	58.3	0.091
Borderline	Reference	/	Reference	/	Reference	/	Reference	/	/	/
High	−0.015 (−0.024 to − 0.005)	0.002	−0.003 (− 0.013 to 0.008)	0.626	−0.016 (− 0.030 to − 0.002)	0.025	−0.011 (− 0.020 to − 0.002)	0.013	43.7	0.169
Memory										
Normal	−0.008 (−0.018 to 0.002)	0.097	0.010 (− 0.002 to 0.021)	0.099	−0.013 (− 0.034 to 0.009)	0.257	−0.003 (− 0.016 to 0.011)	0.715	69.2	0.039
Borderline	Reference	/	Reference	/	Reference	/	Reference	/	/	/
High	−0.006 (− 0.013 to 0.001)	0.076	−0.001 (− 0.008 to 0.006)	0.852	−0.018 (− 0.036 to 0.001)	0.060	−0.005 (− 0.012 to 0.002)	0.138	40.7	0.185
Executive function										
Normal	−0.008 (− 0.018 to 0.002)	0.107	0.007 (−0.005 to 0.019)	0.276	−0.001 (− 0.017 to 0.015)	0.902	−0.001 (− 0.011 to 0.008)	0.777	42.2	0.177
Borderline	Reference	/	Reference	/	Reference	/	Reference	/	/	/
High	−0.003 (− 0.010 to 0.004)	0.404	−0.006 (− 0.014 to 0.001)	0.108	−0.012 (− 0.025 to 0.002)	0.101	−0.005 (− 0.010 to 0.000)	0.031	0.0	0.513
Orientation										
Normal	−0.021 (− 0.039 to − 0.003)	0.025	−0.004 (− 0.025 to 0.016)	0.677	0.011 (−0.006 to 0.029)	0.210	−0.005 (− 0.024 to 0.015)	0.640	67.6	0.046
Borderline	Reference	/	Reference	/	Reference	/	Reference	/	/	/
High	−0.019 (− 0.032 to − 0.007)	0.003	0.004 (−0.009 to 0.016)	0.573	0.002 (−0.013 to 0.017)	0.749	−0.005 (− 0.020 to 0.011)	0.550	73.6	0.023

* After adjusting for age, race (except for the CHARLS), body mass index, education, cohabitation status, current smoking, alcohol consumption, exercise, depressive symptoms, antihypertension medication, hypercholesterolemia, diabetes, coronary heart disease, stroke, cancer, and chronic lung disease

*BP*, blood pressure; *HRS*, the Health and Retirement Study; *ELSA*, the English Longitudinal Study of Ageing; *CHARLS*, the China Health Retirement Longitudinal Study

**Table 3:** Mean differences in rate of cognitive change (SD/year) in women

BP Group	HRS (***n*** = 4154)		ELSA (***n*** = 2996)		CHARLS (***n*** = 2759)		Pooled analysis (***n*** = 9909)			
	***β*** (95% Cl)*	***P*** value	***β*** (95% Cl)*	***P*** value	***β*** (95% Cl)*	***P*** value	Pooled ***β*** (95% Cl)*	***P*** value	***I***^**2**^ (%)	***P*** value
Global										
Normal	−0.002 (−0.013 to 0.009)	0.747	0.017 (0.003 to 0.030)	0.015	0.006 (−0.010 to 0.022)	0.471	0.006 (−0.005 to 0.018)	0.269	54.8	0.109
Borderline	Reference	/	Reference	/	Reference	/	Reference	/	/	/
High	−0.020 (− 0.028 to − 0.012)	< 0.001	−0.022 (− 0.033 to − 0.012)	< 0.001	− 0.004 (− 0.019 to 0.011)	0.612	−0.017 (− 0.026 to − 0.008)	< 0.001	53.6	0.116
Memory										
Normal	−0.004 (− 0.012 to 0.004)	0.358	0.012 (0.003 to 0.022)	0.011	0.016 (−0.005 to 0.037)	0.126	0.007 (−0.006 to 0.020)	0.317	74.9	0.019
Borderline	Reference	/	Reference	/	Reference	/	Reference	/	/	/
High	−0.013 (− 0.019 to − 0.006)	< 0.001	− 0.015 (− 0.022 to − 0.008)	< 0.001	− 0.008 (− 0.027 to 0.011)	0.409	−0.013 (− 0.018 to − 0.009)	< 0.001	0.0	0.726
Executive function										
Normal	0.000 (−0.008 to 0.007)	0.956	0.010 (0.001 to 0.019)	0.031	0.001 (− 0.014 to 0.016)	0.866	0.004 (− 0.003 to 0.011)	0.285	33.2	0.224
Borderline	Reference	/	Reference	/	Reference	/	Reference	/	/	/
High	−0.005 (−0.011 to 0.001)	0.089	−0.009 (− 0.015 to − 0.002)	0.016	0.005 (−0.009 to 0.019)	0.494	−0.005 (− 0.011 to 0.000)	0.069	31.1	0.234
Orientation										
Normal	0.003 (− 0.011 to 0.016)	0.709	0.011 (−0.005 to 0.026)	0.193	−0.004 (− 0.022 to 0.014)	0.671	0.004 (−0.005 to 0.013)	0.439	0.0	0.493
Borderline	Reference	/	Reference	/	Reference	/	Reference	/	/	/
High	−0.016 (− 0.026 to − 0.005)	0.004	−0.022 (− 0.034 to − 0.010)	< 0.001	− 0.006 (− 0.022 to 0.011)	0.513	−0.016 (− 0.024 to − 0.008)	< 0.001	20.6	0.284

* After adjusting for age, race (except for the CHARLS), body mass index, education, cohabitation status, current smoking, alcohol consumption, exercise, depressive symptoms, antihypertension medication, hypercholesterolemia, diabetes, coronary heart disease, stroke, cancer, and chronic lung disease.

*BP*, blood pressure; *HRS*, the Health and Retirement Study; *ELSA*, the English Longitudinal Study of Ageing; *CHARLS*, the China Health Retirement Longitudinal Study

### Sensitivity analyses

**Fig. 2:**
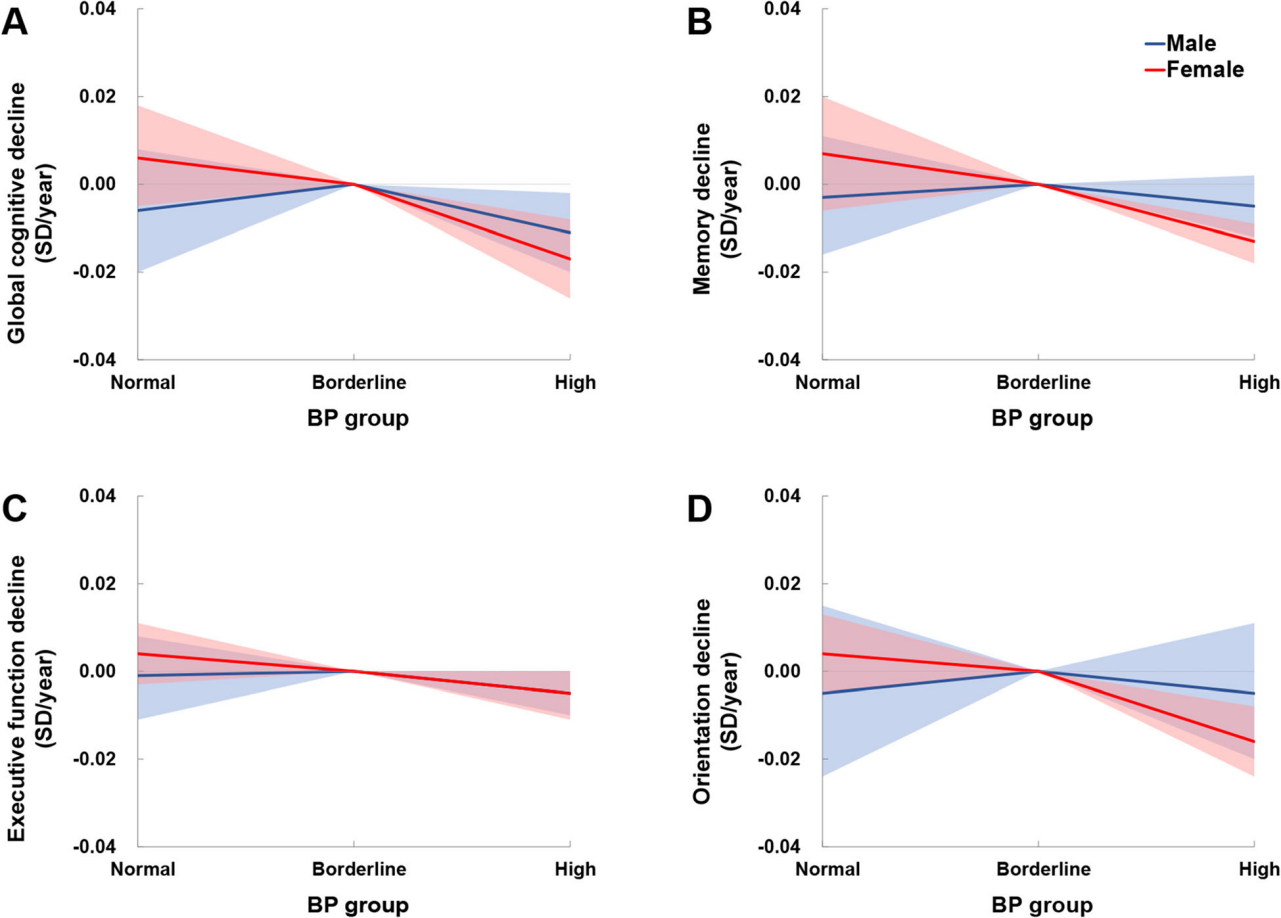
Mean differences in rate of cognitive change by sex. Solid lines represent adjusted mean differences in cognitive change of global cognition (**a**), memory (**b**), executive function (**c**), and orientation (**d**) after adjusting for age, race (except for the CHARLS), BMI, education, cohabitation status, current smoking, alcohol consumption, exercise, depressive symptoms, antihypertension medication, diabetes, hypercholesterolemia, coronary heart disease, stroke, cancer, and chronic lung disease. The shadows represent the 95% CIs. The detailed results are presented in Tables 2 and 3

Sensitivity analyses covered 8102 participants who never used antihypertension medication, 14,620 without hypotension throughout the study, 14,448 without coronary heart disease or stroke at baseline, and 26,360 with BP measurement at ≥ 2 occasions (Additional file 1: Tables S5 and S6). Besides, the results of sensitivity analyses using original cognitive scores are presented in Additional file 1: Tables S7 and S8. The differences in original cognitive score decline rate between BP groups were consistent with those in *Z* scores. No significant difference in cognitive decline rate between the Normal and the Borderline BP groups was observed in any sensitivity analysis (Fig. 2; Additional file 1: Tables S5 to S8).

### Non-response analyses

**Fig. 3:**
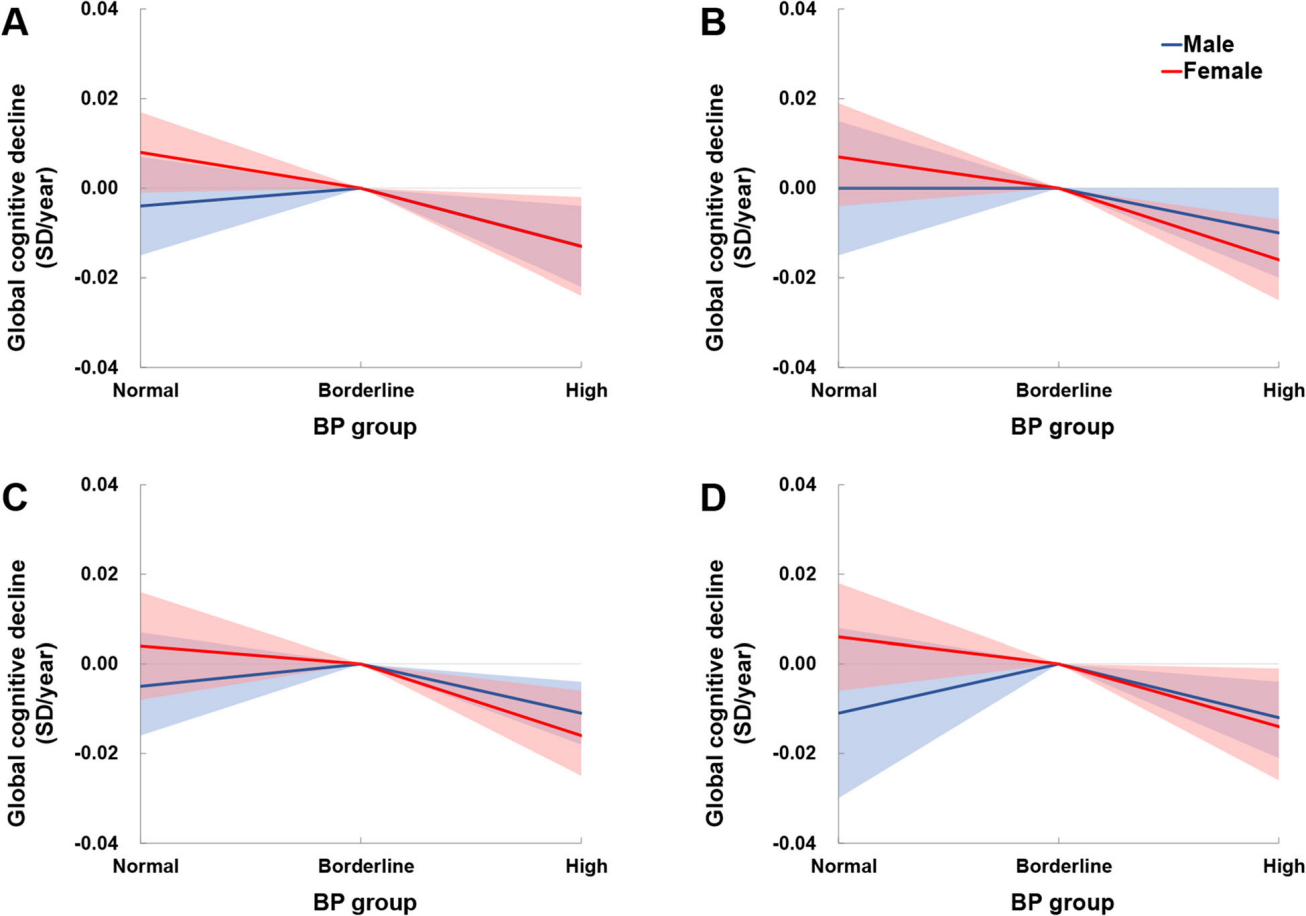
Mean differences in rate of global cognitive change in sensitivity analyses. Solid lines represent adjusted mean differences in global cognition among participants without antihypertension medication using (**a**), hypotension (**b**), coronary heart disease or stroke (**c**), or with ≥ 2 BP measurements (**d**) after adjusting for age, race (except for the CHARLS), BMI, education, cohabitation status, current smoking, alcohol consumption, exercise, depressive symptoms, antihypertension medication, diabetes, hypercholesterolemia, coronary heart disease, stroke, cancer, and chronic lung disease. The shadows represent the 95% CIs. The detailed results are presented in Additional file 1: Tables S4 and S5

A total of 20,700 participants (HRS 7770; ELSA 6329; CHARLS 6601) were excluded for < 3 times BP measurement throughout the study or no follow-up cognitive score (Fig. 3). The excluded participants presented features as follows in all the three cohorts: older, had higher systolic BP, lower level of education, more likely to live alone, less active in physical activity, higher rate of self-reported coronary heart disease, and had lower executive scores (Additional file 1: Table S9).

## Discussion

In this pooled study of the HRS, the ELSA, and the CHARLS with large nationally representative samples, we observed a significantly faster global cognitive decline in the High BP group than that in the Borderline BP group after adjustment, whereas no significant difference in global cognitive decline rate was detected between the Normal and the Borderline BP groups. These findings stand in most single cognitive domains and sensitivity analyses. As far as we know, the present study is one of the first and largest studies to analyze the association between long-term BP and cognitive decline rate regarding the different hypertension thresholds of the ACC/AHA guidelines and the ESC/ESH guidelines.

The findings in this study further contribute to the association between hypertension and cognitive function. A systematic review of 209 prospective studies in 2020 revealed a significant association between midlife hypertension and global cognitive function using low-to-moderate evidence [6]. This review used binary outcomes and did not focus on the association between hypertension and cognitive decline rate [6]. Another systematic review in 2020 collected data from randomized clinical trials and demonstrated a significantly lower risk of dementia or cognitive impairment for individuals using antihypertensive agents compared with control [28]. No significant association between BP lowering and change in cognitive scores was detected in the meta-analysis of eight trials [28]. Consistent with our results, Gottesman et al. reported that midlife hypertension at baseline was associated with faster global cognitive decline in a 20-year cohort [29]. In a 2-year study among individuals with mild cognitive impairment, Goldstein et al. grouped participants using an approach that was partly similar to what we used [30]. They found that cognitive function of individuals with hypertension on two or three occasions declined significantly faster than those with BP < 140/90 mmHg on all three occasions [30]. Noteworthy, all the aforementioned studies defined hypertension as BP ≥ 140/90 mmHg and, except that by Goldstein et al., only used baseline BP. The present study observed a significant difference in global cognitive decline rate between the Borderline and the High BP groups. According to previous studies, a clinically significant change was defined as a decline of 0.5 SD [31, 32]. The 95% CI of global cognitive decline difference between the High and the Borderline BP groups would cover this magnitude in 25 years for men and in 20 years for women. The present study innovatively set the Borderline BP group of individuals with long-term BP under 140/90 mmHg but at least one measurement reaching 130/80 mmHg among all the 3 to 5 occasions. This grouping approach allowed us to address the distinction between hypertension management guidelines and compare individuals with BP under different thresholds. No significant difference in pooled global cognitive decline rate between the Normal and the Borderline BP groups was observed in main or sensitivity analyses. Therefore, among individuals with BP under the threshold of the ESC/ESH guidelines (140/90 mmHg), long-term BP under that of the threshold of the ACC/AHA guidelines (130/80 mmHg) did not make a significant difference in cognitive decline rate.

Remarkably, there have been studies reporting the association between low BP and poorer cognition [6, 33]. An SBP ≤ 128 mmHg has been associated with faster cognitive decline in a cohort [33]. A recent systematic review also suggested that a DBP between 90 and 100 mmHg is an optimum level in older individuals [6]. Thus, we conducted a sensitivity analysis excluding all participants who ever presented hypotension on any BP measurement occasions throughout the study. In participants without hypotension, no significant difference was observed in the global cognitive decline rate between the Normal and the Borderline BP groups. Compared with the Borderline BP group, the High BP group exhibited a marginal significantly faster global cognitive decline in men (pooled *β* = − 0.010 SD/year; 95% CI − 0.020 to 0.000; *P* = 0.058; Additional file 1: Table S4) and significantly in women (pooled *β* = − 0.016 SD/year; 95% CI − 0.025 to − 0.007; *P* < 0.001; Additional file 1: Table S5) in the sensitivity analysis.

Several mechanisms have been indicated responsible for the association between BP and cognitive decline. Cortical white matter lesions and microvascular damage in the brain have been widely accepted as the mediating factors between hypertension and cognition [1, 34, 35]. Hypertension with BP ≥ 140/90 mmHg was associated with increased white matter lesions in a community-based study [34]. And arterial stiffening that was caused by long-standing hypertension might lead to small vessel ischemic disease in the brain [35]. Brain volume reduction has also been indicated as another manifestation of brain damage caused by hypertension in a systematic review [36]. What is more, a review suggested that low BP might cause cerebral hypoperfusion and then accelerate cognitive decline [37].

The foremost strength of the present study is repeated BP measurements during long follow-up. Most previous cohort studies evaluating the association between BP and cognitive decline or dementia only employed the BP at baseline [7, 29, 38–43]. A grouping approach using multiple BP measurements employed has also been adopted in a few studies, which was similar to but distinct from what we used [30, 44]. In the present study, BP measurements on at least three occasions made it possible to evaluate the association between long-term BP maintenance and cognitive decline. BP maintenance might be more clinically relevant than baseline BP for the following two reasons. On the one hand, the association between visit-to-visit variability in BP and cognitive decline has been demonstrated [45, 46]. On the other hand, failed BP maintenance under the hypertension threshold would initiate antihypertensive therapy. Thus, the present work is a useful supplement to previous studies on BP and cognitive decline. Second, the three large nationally representative cohorts from the US, England, and China enhance the certainty and generalizability of results. Third, the primary outcome, the decline rate of global cognitive *Z* score, was calculated from cognitive assessments covering three domains, memory, executive function, and orientation. It would be more sensitive to detect cognitive changes than binary outcomes such as dementia incidence.

This study has several limitations. First, the inherent limitation of observational study restricts this work from confirming a causal relationship. Although there has been substantial evidence from randomized trials supporting the effect of BP levels on cognitive function [6, 28], a reversed causality still could not be completely ruled out. Second, among 38,290 individuals with complete data at baseline, only 17,590 (45.9%) individuals were included in the main analysis, which would introduce selection bias and undermine the generalizability of our findings. Non-response analysis showed that participants who were excluded due to less than three BP measurements or no follow-up cognitive score were older and less healthy. To address this concern, sensitivity analysis among individuals with BP measurement on at least two occasions was conducted. This sensitivity analysis included 26,360 of 38,290 (68.8%) participants with complete baseline data and yielded findings in keeping with that in the main analysis. Third, isolated tests, rather than a comprehensive test, were used to evaluate cognitive function. A subtle cognitive decline might not be detected due to insufficient sensitivity. Although the three cohorts shared the same tests on memory and orientation, the executive function was evaluated using different tests. To be exact, the executive function was assessed by the Serial Sevens test and the counting-backward test in the HRS; the animal fluency test in the ELSA; the Serial Sevens test; and an intersecting pentagon copying test in the CHARLS. *Z* scores were used to evaluate the global cognitive function, while *Z* scores were subjected to various distributions of original cognitive scores, especially those from different executive function tests. Besides, *Z* scores caused difficulty in understanding the difference in cognitive decline rate. Thereby, analyses using the original cognitive scores were conducted. The results in Additional file 1: Tables S7 and S8 are presented as points/year for better understanding. For instance, the memory score in the High BP group would reduce faster by 0.044 points/year compared to that in the Borderline BP group for women, and one point in the memory test stands for a recalled word. Fourth, although a large group of covariates had been measured and adjusted in this study, there were still some unmeasured confounding factors such as *APOE* status and reduced renal function, which might confound the results. Fifth, there was inherent between-cohort heterogeneity, which might come from the significantly different covariates listed in Additional file 1: Table S2, the discrepancy in executive function tests, and other unmeasured factors. Due to considerable (*I*^*2*^ over 75%) between-cohort heterogeneity in the pooled analysis of global cognitive decline, most analyses were conducted by sex, while moderate heterogeneity (*I*^*2*^ between 30% and 60%) persisted [27]. Sixth, white coat hypertension could not be excluded and might introduce measurement bias.

## Conclusions

The Borderline BP group was associated with significantly slower cognitive decline compared with the High BP group but not associated with a significantly different cognitive decline rate compared with the Normal BP group. The findings from this study indicated that it might not be necessary for individuals with borderline BP (between 130/80 and 140/90 mmHg) to initiate antihypertension therapy in consideration of cognitive decline. Prospective observational and interventional studies on the association between long-term BP and cognitive decline are warranted.

## Supplementary Information

**Additional file 1: Supplemental Methods.** Details of cognitive assessment and covariates. **Figure S1.** Mean differences in rate of cognitive change. **Figure S2.** Mean differences in rate of cognitive change by sex with the Normal BP group as reference. **Table S1.** Number and proportion of participants completing planned BP measurement visits. **Table S2.** BP group and covariates of participants in the pooled analysis. **Table S3.** Mean differences in rate of cognitive change (SD/year). **Table S4.** Cognitive decline rate (SD/year) of the High BP group compared to the Normal BP group. **Table S5.** Mean differences in rate of global cognitive change (SD/year) in men: sensitivity analyses. **Table S6.** Mean differences in rate of global cognitive change (SD/year) in women: sensitivity analyses. **Table S7.** Mean differences in rate of cognitive change (points/year) in men: sensitivity analyses. **Table S8.** Mean differences in rate of cognitive change (points/year) in women: sensitivity analyses. **Table S9.** Comparison of baseline characteristics between participants included and excluded.

## Data Availability

Original survey datasets from the HRS, the ELSA, and the CHARLS are freely available to all bonafide researchers. Access to data can be obtained via visiting their websites (https://hrs.isr.umich.edu/data-products, https://beta.ukdataservice.ac.uk/datacatalogue/series/series?id = 200011, and http://charls.pku.edu.cn/pages/data/111/zh-cn.html). The data can also be obtained on request (xiewuxiang@hsc.pku.edu.cn).

## Abbreviations

ACC: American College of Cardiology
AHA: American Heart Association
BMI: Body mass index
BP: Blood pressure
CESD: Center for Epidemiologic Studies Depression
CHARLS: China Health Retirement Longitudinal Study
CI: Confidence interval
ELSA: English Longitudinal Study of Ageing
ESC: European Society of Cardiology
ESH: European Society of Hypertension
HRS: Health and Retirement Study
IQR: Interquartile range
SD: Standard deviation
