# Supplementary material for: Different hypertension thresholds and cognitive decline: a pooled analysis of three ageing cohorts
Source: BMC Med. 2021 Nov 1;19:287. doi: 10.1186/s12916-021-02165-4 (PMC8561998; doi:10.1186/s12916-021-02165-4)
Supplement: Supplementary file 1 — Additional file 1: Supplemental Methods. Details of cognitive assessment and covariates. Figure S1. Mean differences in rate of cognitive change. Figure S2. Mean differences in rate of cognitive change by sex with the Normal BP group as reference. Table S1. Number and proportion of participants completing planned BP measurement visits. Table S2. BP group and covariates of participants in the pooled analysis. Table S3. Mean differences in rate of cognitive change (SD/year). Table S4. Cognitive decline rate (SD/year) of the High BP group compared to the Normal BP group. Table S5. Mean differences in rate of global cognitive change (SD/year) in men: sensitivity analyses. Table S6. Mean differences in rate of global cognitive change (SD/year) in women: sensitivity analyses. Table S7. Mean differences in rate of cognitive change (points/year) in men: sensitivity analyses. Table S8. Mean differences in rate of cognitive change (points/year) in women: sensitivity analyses. Table S9. Comparison of baseline characteristics between participants included and excluded. [file 12916_2021_2165_MOESM1_ESM.docx]

**Additional File 1**

Ma Y, Hua R, Yang Z, Zhong B, Yan L, Xie W. Different hypertension thresholds and cognitive decline: a pooled analysis of three ageing cohorts. *BMC Medicine.*

**Supplemental Methods.** Details of cognitive assessment and covariates.

**Figure S1.** Mean differences in rate of cognitive change.

**Figure S2.** Mean differences in rate of cognitive change by sex with the Normal BP group as reference.

**Table S1.** Number and proportion of participants completing planned BP measurement visits.

**Table S2.** BP group and covariates of participants in the pooled analysis.

**Table S3.** Mean differences in rate of cognitive change (SD/year).

**Table S4.** Cognitive decline rate (SD/year) of the High BP group compared to the Normal BP group.

**Table S5.** Mean differences in rate of global cognitive change (SD/year) in men: sensitivity analyses.

**Table S6.** Mean differences in rate of global cognitive change (SD/year) in women: sensitivity analyses.

**Table S7.** Mean differences in rate of cognitive change (points/year) in men: sensitivity analyses.

**Table S8.** Mean differences in rate of cognitive change (points/year) in women: sensitivity analyses.

**Table S9.** Comparison of baseline characteristics between participants included and excluded.

**Supplemental Methods.** Details of cognitive assessment and covariates.

1. **Cognitive assessment**

Cognitive assessment was conducted in face-to-face interviews and covered three domains: memory, executive function and orientation. The HRS, the ELSA and the CHARLS used the same methods to assess memory and orientation.

- 1. *Memory*

The interviewers read out a set of 10 words “at a slow steady rate approximately one word every 2 seconds” and then ask the individual to “recall aloud as many of the words as you can, in any order”. Enough time was allowed to recall, approximately up to 2 minutes. After questions and tests on other aspects, the participants were again asked to recall the words as many as they could.

- 1. *Executive function*

In the HRS, participants were asked to answer “One hundred minus 7 equals what? And 7 from that? And 7 from that? And 7 from that? And 7 from that?” In addition, participants were asked to count backward as quickly as he or she could from 20. The correct response included counting down from 19 to 10 or from 20 to 11. Participants had two opportunities to perform the counting backwards test.

In the ELSA, participants were asked to “name as many different animals as you can think of” in one minute.

In the CHARLS, participants were asked to answer “What does 100 minus 7 equal? And 7 from that? And 7 from that? And 7 from that? And 7 from that?” In addition, participants were requested to see and draw the following picture.

- 1. *Orientation*

The participants were asked to report the “today's date (the day of month, month, year)” and “what day of the week it is today”.

1. **Covariates**

All the covariates were collected in face-to-face interviews unless otherwise stated.

- 1. *Sex*

Sex were coded with or without asking.

- 1. *Age*

Participants were asked to report “date of birth”.

- 1. *Race*

Participants in the HRS reported the race, which was coded into three groups: white/Caucasian, black/African American, and other. If more than one race was reported, participants were asked which one they consider to be their primary race.

Race in the ELSA was coded into white and non-white.

The CHARLS did not include white person.

- 1. *Body mass index*

Body mass index was calculated with interviewer-performed physical examination and the following formula: weight (kg) / height^2^ (m^2^).

- 1. *Education*

In the HRS, participants were asked to report the years of education. High level of education was defined as 12 or more years of education.

In the ELSA, participants reported their “highest educational qualification” in a self-completion questionnaire, where education level was classified as no qualification, level 1 national vocational qualification (NVQ) or certificate of secondary education, NVQ2 or general certificate of education (GCE) O-level, NVQ3 or GCE A-level, higher qualification but below degree, and degree level or higher or NVQ4/5. High level of education was defined as ≥ NVQ3 or GCE A-level.

In the CHARLS, participants reported their “the highest level of education completed” in interview. Education level was classified as no formal education (illiterate), did not finish primary school but capable of reading and/or writing, sishu/home school, elementary school, middle school, high school, vocational school, two-/three-year college/associate degree, four-year college/Bachelor’s degree, Master’s degree, Doctoral degree/Ph.D. High level of education was defined as ≥ high school.

- 1. *Cohabitation status*

Cohabitation status indicated currently living alone or not. Participants in both cohorts were asked to report their “current legal marital status”. Common-law marriage was considered as married.

In the HRS, participants chose from “married, married (spouse absent), partnered, separated, divorced, separated/divorced, widowed, never married”. Living alone was defined as separated, divorced, separated/divorced, widowed, or never married.

In the ELSA, participants chose from “single (that is never married), married (first and only marriage), a civil partner in a legally-recognized civil partnership, remarried (second or later marriage), legally separated, divorced, and widowed”. Living alone was defined as single, legally separated, divorced, or widowed.

In the CHARLS, participants chose from “married with spouse present, married but not living with spouse temporarily for reasons such as work, separated, divorced, widowed, and never married”. Living alone was defined as separated, divorced, widowed, or never married.

- 1. *Current smoking*

Participants were asked to answer: “Do you smoke cigarettes now?” in the HRS, “Do you smoke cigarettes at all nowadays” in the ELSA or “Do you still chew tobacco, smoke a pipe, smoke self-rolled cigarettes, or smoke cigarettes/cigars?” in the CHARLS with Yes or No.

- 1. *Alcohol consumption*

Participants in the HRS was asked to answer: "Do you ever drink any alcoholic beverages, such as beer, wine, or liquor?" If yes, the follow-up question was asked: "In the last three months, on average, how many days per week have you had any alcohol to drink? (For example, beer, wine, or any drink containing liquor.)"

Participants in the ELSA chose the frequency of alcoholic drinking in the past 12 months in a self-completion questionnaire. Twice a day or more, daily or almost daily, once or twice a week, once or twice a month, special occasions only, or, not at all?

In the CHARLS, alcohol consuming involved the following three questions:

1. How often did you drink liquor, including white liquor, whisky, and others per month in the last year?
2. How many times per month did you drink beer in the last year?
3. How often did you drink wine or rice wine per month in the last year?

The participants were asked to choose one of the following responses for each item above.

1. Once a month
2. 2-3 times a month
3. Once a week
4. 2-3 times a week
5. 4-6 times a week
6. Once a day
7. Twice a day
8. More than twice a day

Alcohol consuming in the CHARLS was defined as at least once a week for whichever of the above three questions.

- 1. *Exercise*

Participants were asked to report their frequencies of vigorous, moderate or light physical sports or activities. Participants who reported at least once per week of vigorous or moderate sports or activities were considered physically active.

- 1. *Depressive symptoms*

Depressive symptoms were evaluated using different versions of the Center for Epidemiologic Studies Depression (CESD) Scale in the three cohorts. The HRS and the ELSA used an eight-item version of the CESD scale. The participants answered the following eight questions with Yes or No for much of the time during the past week.

1. You felt depressed?
2. You felt that everything you did was an effort?
3. Your sleep was restless?
4. You were happy?
5. You felt lonely?
6. You enjoyed life?
7. You felt sad?
8. You could not get going?

The CHARLS instead used a ten-item version of the CESD scale as below.

1. I was bothered by things that don't usually bother me.
2. I had trouble keeping my mind on what I was doing.
3. I felt depressed.
4. I felt everything I did was an effort.
5. I felt hopeful about the future.
6. I felt fearful.
7. My sleep was restless.
8. I was happy.
9. I felt lonely.
10. I could not get "going."

The participants were asked to choose one of the following responses for each item above that happened during the last week.

1. Rarely or none of the time (< 1 day)
2. Some or a little of the time (1-2 days)
3. Occasionally or a moderate amount of the time (3-4 days)
4. Most or all of the time (5-7 days)
   1. *Antihypertension medication*

Participants were asked whether they were taking any medication in order to lower his or her blood pressure at each wave. The proportion of waves reporting any antihypertension medication using in all attending waves was used. For example, a participant attended four waves throughout the study and reported antihypertension medication using at two waves, then the proportion would be 0.5.

- 1. *Diabetes, hypercholesterolemia, coronary heart disease, stroke, cancer, asthma, and chronic lung disease*

Participants were requested to confirm whether they had been told by a doctor that they had the following conditions:

1. diabetes (diabetes or high blood sugar),
2. hypercholesterolemia,
3. heart disease (heart attack, coronary heart disease, angina, congestive heart failure, or other heart problems in the HRS and the CHARLS; angina or heart attack in the ELSA),
4. stroke,
5. cancer (cancer or malignant tumour),
6. asthma,
7. chronic lung disease (such as chronic bronchitis or emphysema).

In addition, confirmation of current anti-diabetic therapy was included in the definition of diabetes.

- 1. *Hypercholesterolemia*

Hypercholesterolemia was defined as self-reported physician-diagnosed hypercholesterolemia, or self-reported use of lipid-lowering medication, or total cholesterol ≥240 mg/dL[1]. Participants were requested to report whether taking medication to lower cholesterol in wave 8 of the HRS, wave 1 of the ELSA, and the wave1 of the CHARLS. In addition, individuals with serum total cholesterol (TC) ≥240 mg/dL was also regarded as having hypercholesterolemia. TC was examined in wave 1 of the CHARLS. Since the ELSA did not report TC at wave 1, TC in wave 2 of the ELSA were used instead. The HRS only took blood samples from half of the participants at a wave and the other half at next wave. Thus, TC in the wave 8 and wave 9 of the HRS were used.

**References:**

1. Carroll MD, Fryar CD, Nguyen DT: Total and High-density Lipoprotein Cholesterol in Adults: United States, 2015-2016. *NCHS Data Brief* 2017(290):1-8.

**Supplemental Figures**


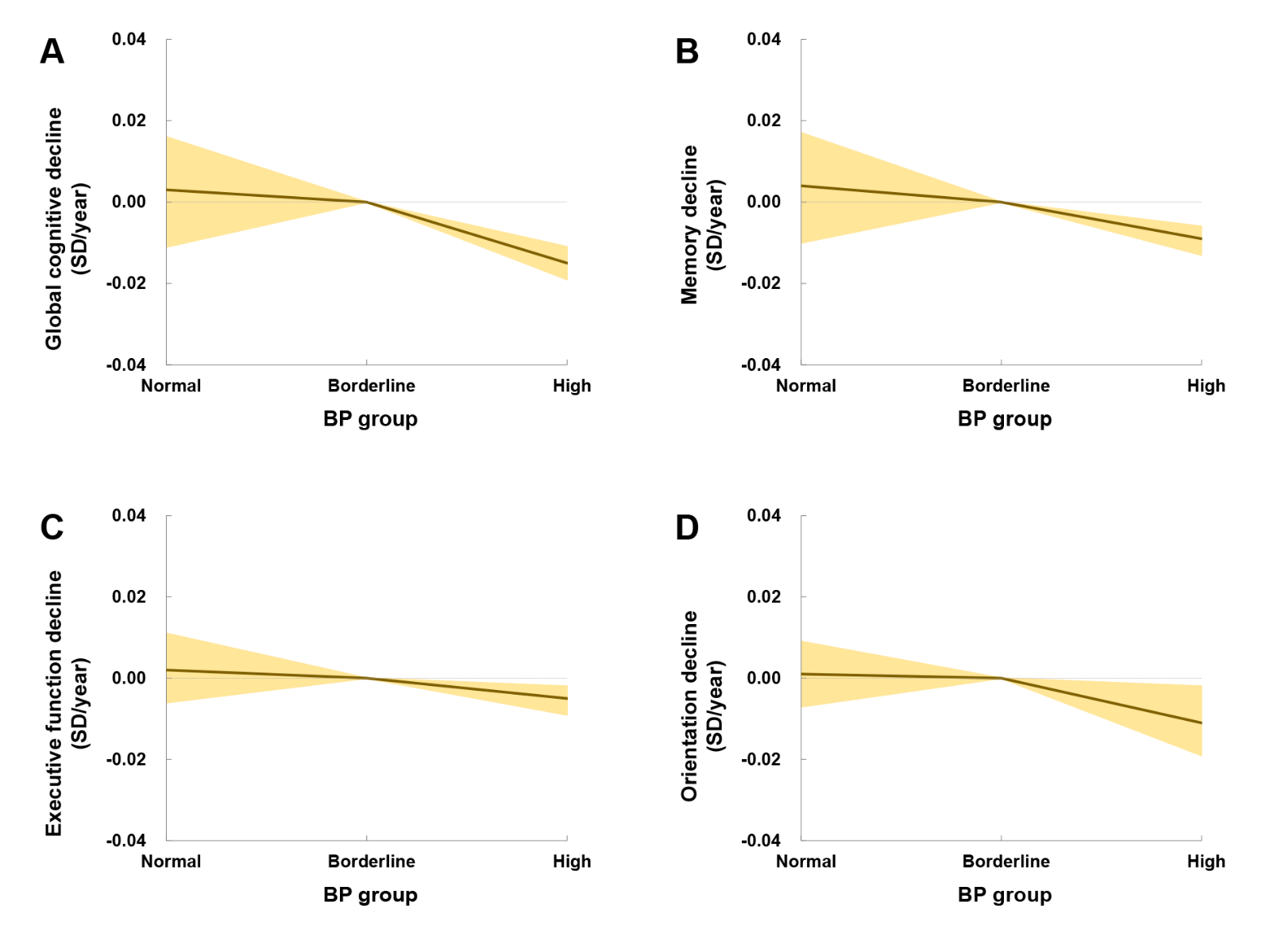


**Figure S1.** Mean differences in rate of cognitive change.

Solid lines represent adjusted mean differences in cognitive change of global cognition(a), memory (b), executive function (c), and orientation (d) after adjusting for age, race (except for the CHARLS), BMI, education, cohabitation status, current smoking, alcohol consumption, exercise, depressive symptoms, antihypertension medication, diabetes, hypercholesterolemia, coronary heart disease, stroke, cancer, and chronic lung disease. The shadows represent the 95% CIs. The detailed results are presented in Additional file: Tables S3.


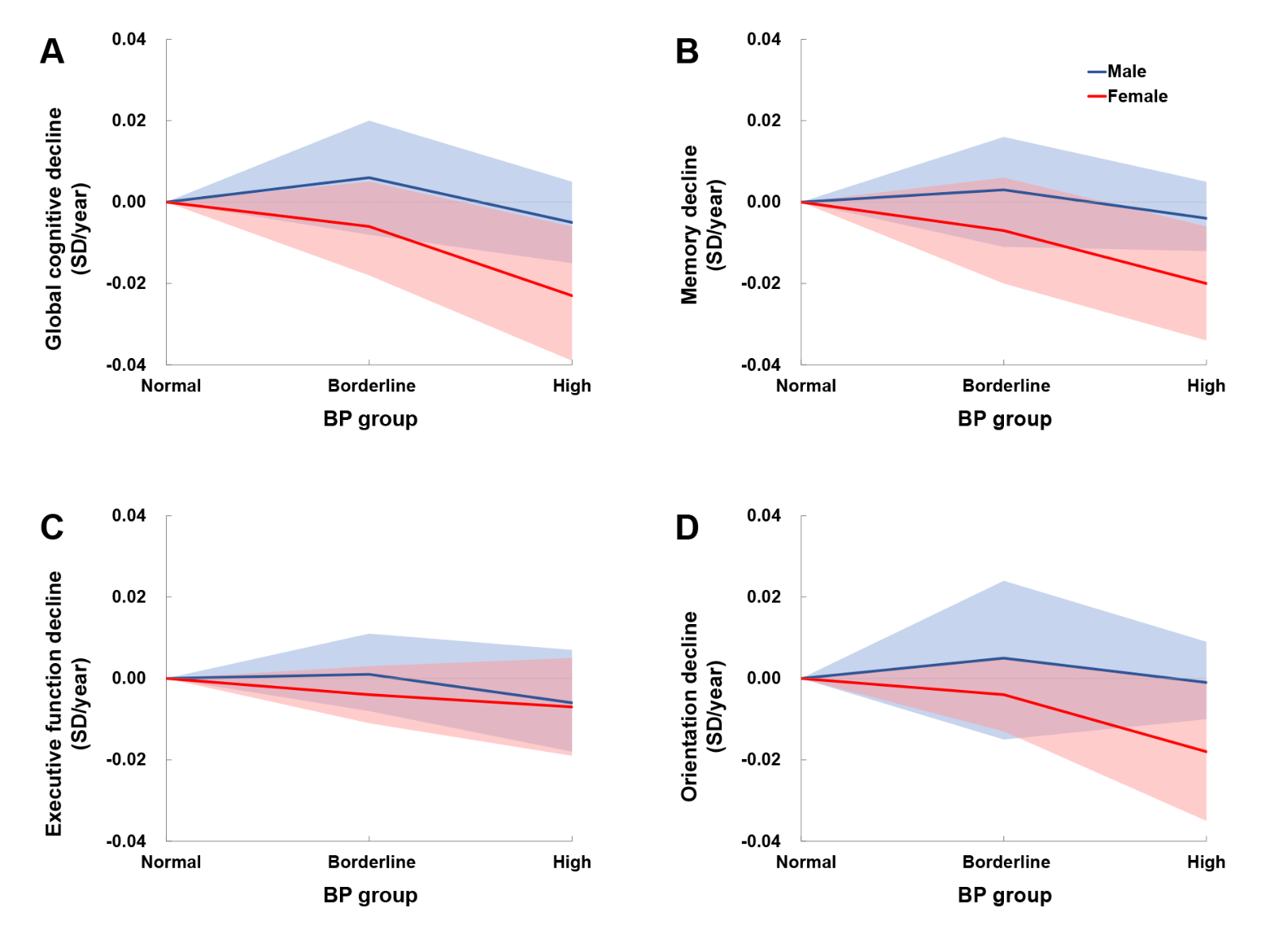


**Figure S2.** Mean differences in rate of cognitive change by sex with the Normal BP group as reference.

Solid lines represent adjusted mean differences in cognitive change of global cognition(a), memory (b), executive function (c), and orientation (d) after adjusting for age, race (except for the CHARLS), BMI, education, cohabitation status, current smoking, alcohol consumption, exercise, depressive symptoms, antihypertension medication, diabetes, hypercholesterolemia, coronary heart disease, stroke, cancer, and chronic lung disease. The shadows represent the 95% CIs. The detailed cognitive decline rate (SD/year) of High BP group compared to Normal BP group are presented in Additional file: Table S6.

**Supplemental Tables**

**T****able S1.** Number and proportion of participants completing planned BP measurement visits.

| **Planned BP measurement visit^*^** | **HRS** | **ELSA** | **CHARLS** |
| --- | --- | --- | --- |
| Visit 1 | 6768 (97.2) | 4655 (87.3) | 5292(100.0) |
| Visit 2 | 6743 (96.8) | 5029 (94.3) | 5292(100.0) |
| Visit 3 | 6851 (98.4) | 5040 (94.5) | 5292(100.0) |
| Visit 4 | 2909 (41.8) | 4426 (83.0) | - |
| Visit 5 | - | 2476 (46.4) | - |

* Data are presented as n (%).

For half participants in the HRS, 4 blood pressure visits were implemented at wave 8, 10, 12, and 14, while the rest participants had their blood pressure measured at wave 9, 11, 13 and 14. For participants in the ELSA, a total of 5 blood pressure measurement visits were conducted at wave 0, 2, 4, 6, and 8, respectively. Participants in the CHARLS had their blood pressure measured at wave 1, 2, and 3.

**Table S2.** BP group and covariates of participants in the pooled analysis.

| **Characteristic** | **Men (n = 7681)** | **Women (n = 9909)** | ***P* value*** | **HRS**  **(n = 6964)** | **ELSA**  **(n = 5334)** | **CHARLS**  **(n = 5292)** | ***P* value^†^** |
| --- | --- | --- | --- | --- | --- | --- | --- |
| Age (years) | 62.8±8.9 | 62.5±9.4 | 0.042 | 66.3±8.0 | 62.4±8.9 | 58.1±8.8 | <0.001 |
| White (%) | 4667 (60.8) | 6315 (63.7) | <0.001 | 5745 (82.5) | 5237 (98.2) | 0 (0) | <0.001 |
| BP group (%) |  |  | <0.001 |  |  |  | <0.001 |
| Normal | 1162 (15.1) | 1951 (19.7) |  | 1114 (16.0) | 581 (10.9) | 1418 (26.8) |  |
| Borderline | 1776 (23.1) | 2158 (21.8) |  | 1744 (25.0) | 962 (18.0) | 1228 (23.2) |  |
| High | 4743 (61.7) | 5800 (58.5) |  | 4106 (59.0) | 3791 (71.1) | 2646 (50.0) |  |
| BMI (kg/m^2^) | 26.7±4.9 | 27.4±5.6 | <0.001 | 29.5±5.6 | 27.5±4.5 | 23.7±3.7 | <0.001 |
| Systolic blood pressure (mmHg) | 134.8±19.4 | 132.6±21.1 | <0.001 | 130.4±19.3 | 140.7±19.1 | 129.6±20.7 | <0.001 |
| Diastolic blood pressure (mmHg) | 79.1±11.9 | 76.4±11.8 | <0.001 | 79.8±11.2 | 77.8±12.0 | 75.8±12.0 | <0.001 |
| High level of education (%) | 3756 (48.9) | 4409 (44.5) | <0.001 | 5710 (82.0) | 1875 (35.2) | 580 (11.0) | <0.001 |
| Living alone (%) | 1154 (15.0) | 2960 (29.9) | <0.001 | 1990 (28.6) | 1592 (29.8) | 532 (10.1) | <0.001 |
| Current smoking (%) | 2543 (33.1) | 1165 (11.8) | <0.001 | 817 (11.7) | 822 (15.4) | 2069 (39.1) | <0.001 |
| Alcohol consumption (%) | 4235 (55.1) | 3020 (30.5) | <0.001 | 2567 (36.9) | 3335 (62.5) | 1353 (25.6) | <0.001 |
| Physically active (%) | 5212 (67.9) | 6401 (64.6) | <0.001 | 5560 (79.8) | 4424 (82.9) | 1629 (30.8) | <0.001 |
| Depressive symptoms (%) | 899 (11.7) | 1900 (19.2) | <0.001 | 834 (12.0) | 707 (13.3) | 1258 (23.8) | <0.001 |
| Taking antihypertension medication (%) | 3758 (50.9) | 5025 (52.9) | 0.009 | 4940 (70.9) | 2715 (50.9) | 1128 (24.6) | <0.001 |
| History of diseases |  |  |  |  |  |  |  |
| Hypertension (%) | 3512 (45.7) | 4638 (46.8) | 0.153 | 4231 (60.8) | 1852 (34.7) | 2067 (39.1) | <0.001 |
| Diabetes (%) | 809 (10.5) | 969 (9.8) | 0.100 | 1193 (17.1) | 294 (5.5) | 291 (5.5) | <0.001 |
| Hypercholesterolemia (%) | 2556 (33.3) | 3251 (32.8) | 0.512 | 2999 (43.1) | 1961 (36.8) | 847 (16.0) | <0.001 |
| Heart disease (%) | 1350 (17.6) | 1492 (15.1) | <0.001 | 1389 (19.9) | 874 (16.4) | 579 (10.9) | <0.001 |
| Stroke (%) | 238 (3.1) | 246 (2.5) | 0.013 | 261 (3.7) | 138 (2.6) | 85 (1.6) | <0.001 |
| Cancer (%) | 495 (6.4) | 702 (7.1) | 0.095 | 858 (12.3) | 294 (5.5) | 45 (0.9) | <0.001 |
| Chronic lung disease (%) | 558 (7.3) | 727 (7.3) | 0.855 | 518 (7.4) | 257 (4.8) | 510 (9.6) | <0.001 |
| Cognitive scores^‡^ |  |  |  |  |  |  |  |
| Memory | 11.7±4.5 | 11.9±4.2 | 0.004 | 10.6±3.2 | 10.3±3.2 | 15.1±4.8 | <0.001 |
| Orientation | 4 (3–4) | 4 (3–4) | 0.142 | 4 (4–4) | 4 (4–4) | 4 (3–4) | <0.001 |

Data are presented as mean ± SD, n (%), or median (IQR).

* *P* value for differences between men and women.

^†^ *P* value for differences among the three cohorts.

^‡^ The executive scores are omitted due to different assessment methods.

**Table S3.** Mean differences in rate of cognitive change (SD/year).

| **BP Group** | **HRS (n = 6964)** | |  | **ELSA (n = 5334)** | |  | **CHARLS (n = 5292)** | |  | **Pooled analysis (n = 17 590)** | | | |
| --- | --- | --- | --- | --- | --- | --- | --- | --- | --- | --- | --- | --- | --- |
|  | **β (95% Cl)*** | ***P* value** |  | **β (95% Cl)*** | ***P* value** |  | **β (95% Cl)*** | ***P* value** |  | **Pooled β (95% Cl)*** | ***P* value** | ***I*^2^ (%)** | ***P* value** |
| **Global** |  |  |  |  |  |  |  |  |  |  |  |  |  |
| Normal | -0.008 (-0.016 to 0.001) | 0.075 |  | 0.015 (0.004 to 0.025) | 0.007 |  | 0.002 (-0.009 to 0.014) | 0.706 |  | 0.003 (-0.011 to 0.016) | 0.686 | 80.8 | 0.005 |
| Borderline | Reference | / |  | Reference | / |  | Reference | / |  | Reference | / | / | / |
| High | -0.018 (-0.024 to -0.012) | <0.001 |  | -0.013 (-0.021 to -0.006) | <0.001 |  | -0.010 (-0.020 to 0.000) | 0.058 |  | -0.015 (-0.019 to -0.011) | <0.001 | 0.0 | 0.400 |
| **Memory** |  |  |  |  |  |  |  |  |  |  |  |  |  |
| Normal | -0.005 (-0.012 to 0.001) | 0.086 |  | 0.013 (0.006 to 0.020) | <0.001 |  | 0.004 (-0.011 to 0.019) | 0.612 |  | 0.004 (-0.010 to 0.017) | 0.580 | 86.6 | 0.001 |
| Borderline | Reference | / |  | Reference | / |  | Reference | / |  | Reference | / | / | / |
| High | -0.010 (-0.014 to -0.005) | <0.001 |  | -0.008 (-0.013 to -0.003) | 0.001 |  | -0.013 (-0.026 to 0.000) | 0.057 |  | -0.009 (-0.013 to -0.006) | <0.001 | 0.0 | 0.798 |
| **Executive function** |  |  |  |  |  |  |  |  |  |  |  |  |  |
| Normal | -0.003 (-0.009 to 0.003) | 0.322 |  | 0.009 (0.002 to 0.017) | 0.012 |  | 0.001 (-0.010 to 0.012) | 0.851 |  | 0.002 (-0.006 to 0.011) | 0.569 | 69.7 | 0.037 |
| Borderline | Reference | / |  | Reference | / |  | Reference | / |  | Reference | / | / | / |
| High | -0.004 (-0.009 to 0.000) | 0.072 |  | -0.008 (-0.013 to -0.002) | 0.004 |  | -0.004 (-0.013 to 0.006) | 0.475 |  | -0.005 (-0.009 to -0.002) | 0.001 | 0.0 | 0.569 |
| **Orientation** |  |  |  |  |  |  |  |  |  |  |  |  |  |
| Normal | -0.006 (-0.017 to 0.005) | 0.308 |  | 0.007 (-0.005 to 0.019) | 0.268 |  | 0.003 (-0.010 to 0.016) | 0.649 |  | 0.001 (-0.007 to 0.009) | 0.816 | 17.4 | 0.298 |
| Borderline | Reference | / |  | Reference | / |  | Reference | / |  | Reference | / | / | / |
| High | -0.017 (-0.025 to -0.009) | <0.001 |  | -0.010 (-0.019 to -0.002) | 0.019 |  | -0.002 (-0.013 to 0.009) | 0.748 |  | -0.011 (-0.019 to -0.002) | 0.013 | 58.1 | 0.092 |

* After adjusting for sex, age, race (except for the CHARLS), body mass index, education, cohabitation status, current smoking, alcohol consumption, exercise, depressive symptoms, antihypertension medication, hypercholesterolemia, diabetes, coronary heart disease, stroke, cancer, and chronic lung disease.

**Table S4.** Cognitive decline rate (SD/year) of the High BP group compared to the Normal BP group.

| **BP Group** | **HRS** | |  | **ELSA** | |  | **CHARLS** | |  | **Pooled analysis** | | | |
| --- | --- | --- | --- | --- | --- | --- | --- | --- | --- | --- | --- | --- | --- |
|  | **β (95% Cl)*** | ***P* value** |  | **β (95% Cl)*** | ***P* value** |  | **β (95% Cl)*** | ***P* value** |  | **Pooled β (95% Cl)*** | ***P* value** | ***I*^2^ (%)** | ***P* value** |
| **Men** | n = 1732 |  |  | n = 1692 |  |  | n = 1319 |  |  | n = 4743 |  |  |  |
| Global | 0.003 (-0.009 to 0.016) | 0.578 |  | -0.008 (-0.023 to 0.007) | 0.312 |  | -0.013 (-0.027 to 0.002) | 0.082 |  | -0.005 (-0.015 to 0.005) | 0.326 | 35.8 | 0.210 |
| Memory | 0.002 (-0.007 to 0.011) | 0.636 |  | -0.010 (-0.020 to 0.000) | 0.046 |  | -0.005 (-0.024 to 0.014) | 0.585 |  | -0.004 (-0.012 to 0.005) | 0.371 | 39.6 | 0.191 |
| Executive function | 0.005 (-0.004 to 0.014) | 0.249 |  | -0.013 (-0.024 to -0.002) | 0.019 |  | -0.011 (-0.025 to 0.003) | 0.139 |  | -0.006 (-0.018 to 0.007) | 0.382 | 74.1 | 0.021 |
| Orientation | 0.002 (-0.015 to 0.018) | 0.849 |  | 0.008 (-0.010 to 0.026) | 0.390 |  | -0.009 (-0.024 to 0.006) | 0.257 |  | -0.001 (-0.010 to 0.009) | 0.889 | 2.1 | 0.360 |
| **Women** | n = 2374 |  |  | n = 2099 |  |  | n = 1327 |  |  | n = 5800 |  |  |  |
| Global | -0.018 (-0.028 to -0.009) | <0.001 |  | -0.039 (-0.050 to -0.028) | <0.001 |  | -0.010 (-0.023 to 0.004) | 0.154 |  | -0.023 (-0.039 to -0.006) | 0.007 | 84.5 | 0.002 |
| Memory | -0.009 (-0.016 to -0.002) | 0.013 |  | -0.028 (-0.035 to -0.020) | <0.001 |  | -0.024 (-0.042 to -0.007) | 0.006 |  | -0.020 (-0.034 to -0.006) | 0.006 | 84.9 | 0.001 |
| Executive function | -0.005 (-0.012 to 0.002) | 0.152 |  | -0.019 (-0.026 to -0.011) | <0.001 |  | 0.004 (-0.009 to 0.016) | 0.577 |  | -0.007 (-0.019 to 0.005) | 0.226 | 83.1 | 0.003 |
| Orientation | -0.018 (-0.031 to -0.006) | 0.003 |  | -0.033 (-0.046 to -0.020) | <0.001 |  | -0.002 (-0.017 to 0.014) | 0.832 |  | -0.018 (-0.035 to -0.001) | 0.034 | 78.6 | 0.009 |

* After adjusting for age, race (except for the CHARLS), body mass index, education, cohabitation status, current smoking, alcohol consumption, exercise, depressive symptoms, antihypertension medication, hypercholesterolemia, diabetes, coronary heart disease, stroke, cancer, and chronic lung disease.

**Table S5.** Mean differences in rate of global cognitive change (SD/year) in men: sensitivity analyses.

| **BP Group** | **HRS** | |  | **ELSA** | |  | **CHARLS** | |  | **Pooled analysis** | | | |
| --- | --- | --- | --- | --- | --- | --- | --- | --- | --- | --- | --- | --- | --- |
|  | **β (95% Cl)*** | ***P* value** |  | **β (95% Cl)*** | ***P* value** |  | **β (95% Cl)*** | ***P* value** |  | **Pooled β (95% Cl)*** | ***P* value** | ***I*^2^ (%)** | ***P* value** |
| **Never used antihypertension medication** | n = 828 |  |  | n = 1135 |  |  | n = 1666 |  |  | n = 3629 |  |  |  |
| Normal | -0.015 (-0.035 to 0.004) | 0.124 |  | 0.004 (-0.014 to 0.023) | 0.640 |  | -0.002 (-0.020 to 0.016) | 0.823 |  | -0.004 (-0.015 to 0.007) | 0.488 | 6.1 | 0.345 |
| Borderline | Reference | / |  | Reference | / |  | Reference | / |  | Reference | / | / | / |
| High | -0.022 (-0.038 to -0.005) | 0.011 |  | -0.007 (-0.020 to 0.007) | 0.327 |  | -0.016 (-0.034 to 0.001) | 0.072 |  | -0.013 (-0.022 to -0.004) | 0.004 | 3.2 | 0.356 |
| **No hypotension** | n = 2463 |  |  | n = 1819 |  |  | n = 2120 |  |  | n = 6402 |  |  |  |
| Normal | -0.013 (-0.028 to 0.003) | 0.120 |  | 0.012 (-0.007 to 0.032) | 0.215 |  | 0.003 (-0.016 to 0.022) | 0.760 |  | 0.000 (-0.015 to 0.015) | 0.999 | 50.1 | 0.135 |
| Borderline | Reference | / |  | Reference | / |  | Reference | / |  | Reference | / | / | / |
| High | -0.015 (-0.025 to -0.005) | 0.003 |  | 0.000 (-0.011 to 0.011) | 0.984 |  | -0.016 (-0.031 to -0.001) | 0.038 |  | -0.010 (-0.020 to 0.000) | 0.058 | 56.1 | 0.103 |
| **No coronary heart disease or stroke** | n = 2064 |  |  | n = 1869 |  |  | n = 2261 |  |  | n = 6194 |  |  |  |
| Normal | -0.015 (-0.032 to 0.001) | 0.066 |  | 0.000 (-0.018 to 0.018) | 0.976 |  | 0.003 (-0.015 to 0.020) | 0.776 |  | -0.005 (-0.016 to 0.007) | 0.422 | 22.4 | 0.276 |
| Borderline | Reference | / |  | Reference | / |  | Reference | / |  | Reference | / | / | / |
| High | -0.013 (-0.023 to -0.002) | 0.020 |  | -0.008 (-0.019 to 0.003) | 0.170 |  | -0.012 (-0.027 to 0.003) | 0.113 |  | -0.011 (-0.018 to -0.004) | 0.002 | 0.0 | 0.806 |
| **BP measurement at ≥ 2 occasions** | n = 4049 |  |  | n = 3419 |  |  | n = 4126 |  |  | n = 11594 |  |  |  |
| Normal | -0.029 (-0.041 to -0.017) | <0.001 |  | 0.004 (-0.010 to 0.018) | 0.587 |  | -0.007 (-0.020 to 0.005) | 0.261 |  | -0.011 (-0.030 to 0.008) | 0.256 | 84.8 | 0.001 |
| Borderline | Reference | / |  | Reference | / |  | Reference | / |  | Reference | / | / | / |
| High | -0.015 (-0.024 to -0.007) | <0.001 |  | -0.004 (-0.013 to 0.005) | 0.385 |  | -0.019 (-0.030 to -0.008) | 0.001 |  | -0.012 (-0.021 to -0.004) | 0.006 | 59.8 | 0.083 |

* After adjusting for age, race (except for the CHARLS), body mass index, education, cohabitation status, current smoking, alcohol consumption, exercise, depressive symptoms, antihypertension medication, hypercholesterolemia, diabetes, coronary heart disease, stroke, cancer, and chronic lung disease.

HRS, the Health and Retirement Study; ELSA, the English Longitudinal Study of Ageing; CHARLS, the China Health Retirement Longitudinal Study.

**Table S6.** Mean differences in rate of global cognitive change (SD/year) in women: sensitivity analyses.

| **BP Group** | **HRS** | |  | **ELSA** | |  | **CHARLS** | |  | **Pooled analysis** | | | |
| --- | --- | --- | --- | --- | --- | --- | --- | --- | --- | --- | --- | --- | --- |
|  | **β (95% Cl)*** | ***P* value** |  | **β (95% Cl)*** | ***P* value** |  | **β (95% Cl)*** | ***P* value** |  | **Pooled β (95% Cl)*** | ***P* value** | ***I*^2^ (%)** | ***P* value** |
| **Never used antihypertension medication** | n = 1196 |  |  | n = 1484 |  |  | n = 1793 |  |  | n = 4473 |  |  |  |
| Normal | 0.000 (-0.015 to 0.015) | 0.958 |  | 0.015 (0.001 to 0.028) | 0.033 |  | 0.008 (-0.010 to 0.026) | 0.411 |  | 0.008 (-0.001 to 0.017) | 0.071 | 0.0 | 0.379 |
| Borderline | Reference | / |  | Reference | / |  | Reference | / |  | Reference | / | / | / |
| High | -0.015 (-0.030 to -0.001) | 0.040 |  | -0.019 (-0.031 to -0.007) | 0.002 |  | 0.001 (-0.018 to 0.021) | 0.886 |  | -0.013 (-0.024 to -0.002) | 0.019 | 38.4 | 0.197 |
| **No hypotension** | n = 3652 |  |  | n = 2289 |  |  | n = 2277 |  |  | n = 8218 |  |  |  |
| Normal | -0.001 (-0.013 to 0.011) | 0.830 |  | 0.017 (0.001 to 0.034) | 0.036 |  | 0.009 (-0.010 to 0.027) | 0.365 |  | 0.007 (-0.004 to 0.019) | 0.230 | 41.0 | 0.184 |
| Borderline | Reference | / |  | Reference | / |  | Reference | / |  | Reference | / | / | / |
| High | -0.020 (-0.029 to -0.012) | <0.001 |  | -0.019 (-0.030 to -0.007) | 0.001 |  | -0.003 (-0.019 to 0.013) | 0.681 |  | -0.016 (-0.025 to -0.007) | <0.001 | 44.0 | 0.168 |
| **No coronary heart disease or stroke** | n = 3366 |  |  | n = 2507 |  |  | n = 2381 |  |  | n = 8254 |  |  |  |
| Normal | -0.006 (-0.017 to 0.006) | 0.331 |  | 0.013 (-0.001 to 0.027) | 0.065 |  | 0.006 (-0.012 to 0.023) | 0.527 |  | 0.004 (-0.008 to 0.016) | 0.545 | 53.9 | 0.114 |
| Borderline | Reference | / |  | Reference | / |  | Reference | / |  | Reference | / | / | / |
| High | -0.017 (-0.026 to -0.009) | <0.001 |  | -0.022 (-0.033 to -0.011) | <0.001 |  | -0.002 (-0.018 to 0.014) | 0.796 |  | -0.016 (-0.025 to -0.006) | 0.001 | 51.8 | 0.126 |
| **BP measurement at ≥ 2 occasions** | n = 5950 |  |  | n = 4319 |  |  | n = 4497 |  |  | n = 14766 |  |  |  |
| Normal | -0.004 (-0.013 to 0.006) | 0.454 |  | 0.016 (0.004 to 0.027) | 0.008 |  | 0.008 (-0.005 to 0.020) | 0.238 |  | 0.006 (-0.006 to 0.018) | 0.304 | 69.7 | 0.037 |
| Borderline | Reference | / |  | Reference | / |  | Reference | / |  | Reference | / | / | / |
| High | -0.019 (-0.026 to -0.011) | <0.001 |  | -0.022 (-0.031 to -0.013) | <0.001 |  | 0.002 (-0.010 to 0.014) | 0.783 |  | -0.014 (-0.026 to -0.001) | 0.029 | 81.3 | 0.005 |

* After adjusting for age, race (except for the CHARLS), body mass index, education, cohabitation status, current smoking, alcohol consumption, exercise, depressive symptoms, antihypertension medication, hypercholesterolemia, diabetes, coronary heart disease, stroke, cancer, and chronic lung disease.

**Table S7.** Mean differences in rate of cognitive change (points/year) in men: sensitivity analyses.

| **BP Group** | **HRS (n = 6964)** | |  | **ELSA (n = 5334)** | |  | **CHARLS (n = 5292)** | |  | **Pooled analysis (n = 17 590)** | | | |
| --- | --- | --- | --- | --- | --- | --- | --- | --- | --- | --- | --- | --- | --- |
|  | **β (95% Cl)*** | ***P* value** |  | **β (95% Cl)*** | ***P* value** |  | **β (95% Cl)*** | ***P* value** |  | **Pooled β (95% Cl)***^†^ | ***P* value** | ***I*^2^ (%)** | ***P* value** |
| **Memory** |  |  |  |  |  |  |  |  |  |  |  |  |  |
| Normal | -0.027 (-0.058 to 0.005) | 0.097 |  | 0.031 (-0.006 to 0.068) | 0.099 |  | -0.061 (-0.165 to 0.044) | 0.257 |  | -0.008 (-0.058 to 0.041) | 0.739 | 69.6 | 0.037 |
| Borderline | Reference | / |  | Reference | / |  | Reference | / |  | Reference | / | / | / |
| High | -0.020 (-0.042 to 0.002) | 0.076 |  | -0.002 (-0.025 to 0.021) | 0.852 |  | -0.086 (-0.175 to 0.003) | 0.060 |  | -0.016 (-0.041 to 0.008) | 0.196 | 47.4 | 0.149 |
| **Executive function** |  |  |  |  |  |  |  |  |  |  |  |  |  |
| Normal | -0.011 (-0.024 to 0.002) | 0.107 |  | 0.042 (-0.034 to 0.118) | 0.276 |  | -0.003 (-0.045 to 0.040) | 0.902 |  | - | - | - | - |
| Borderline | Reference | / |  | Reference | / |  | Reference | / |  | - | - | - | - |
| High | -0.004 (-0.013 to 0.005) | 0.404 |  | -0.038 (-0.085 to 0.008) | 0.108 |  | -0.030 (-0.066 to 0.006) | 0.101 |  | - | - | - | - |
| **Orientation** |  |  |  |  |  |  |  |  |  |  |  |  |  |
| Normal | -0.010 (-0.018 to -0.001) | 0.025 |  | -0.002 (-0.011 to 0.007) | 0.677 |  | 0.012 (-0.006 to 0.030) | 0.210 |  | -0.002 (-0.012 to 0.007) | 0.623 | 57.5 | 0.095 |
| Borderline | Reference | / |  | Reference | / |  | Reference | / |  | Reference | / | / | / |
| High | -0.009 (-0.015 to -0.003) | 0.003 |  | 0.002 (-0.004 to 0.007) | 0.573 |  | 0.003 (-0.013 to 0.018) | 0.749 |  | -0.002 (-0.011 to 0.006) | 0.564 | 70.6 | 0.033 |

* After adjusting for sex, age, race (except for the CHARLS), body mass index, education, cohabitation status, current smoking, alcohol consumption, exercise, depressive symptoms, antihypertension medication, hypercholesterolemia, diabetes, coronary heart disease, stroke, cancer, and chronic lung disease.

^†^ The executive function scores were not pooled due to different tests.

**Table S8.** Mean differences in rate of cognitive change (points/year) in women: sensitivity analyses.

| **BP Group** | **HRS (n = 6964)** | |  | **ELSA (n = 5334)** | |  | **CHARLS (n = 5292)** | |  | **Pooled analysis (n = 17 590)** | | | |
| --- | --- | --- | --- | --- | --- | --- | --- | --- | --- | --- | --- | --- | --- |
|  | **β (95% Cl)*** | ***P* value** |  | **β (95% Cl)*** | ***P* value** |  | **β (95% Cl)*** | ***P* value** |  | **Pooled β (95% Cl)*^†^** | ***P* value** | ***I*^2^ (%)** | ***P* value** |
| **Memory** |  |  |  |  |  |  |  |  |  |  |  |  |  |
| Normal | -0.012 (-0.037 to 0.013) | 0.358 |  | 0.040 (0.009 to 0.070) | 0.011 |  | 0.078 (-0.022 to 0.179) | 0.126 |  | 0.023 (-0.024 to 0.070) | 0.333 | 76.0 | 0.016 |
| Borderline | Reference | / |  | Reference | / |  | Reference | / |  | Reference | / | / | / |
| High | -0.040 (-0.059 to -0.020) | <0.001 |  | -0.049 (-0.072 to -0.026) | <0.001 |  | -0.039 (-0.132 to 0.054) | 0.409 |  | -0.044 (-0.058 to -0.029) | <0.001 | 0.0 | 0.814 |
| **Executive function** |  |  |  |  |  |  |  |  |  |  |  |  |  |
| Normal | 0.000 (-0.010 to 0.010) | 0.956 |  | 0.062 (0.006 to 0.119) | 0.031 |  | 0.003 (-0.035 to 0.042) | 0.866 |  | - | - | - | - |
| Borderline | Reference | / |  | Reference | / |  | Reference | / |  | - | - | - | - |
| High | -0.007 (-0.015 to 0.001) | 0.089 |  | -0.053 (-0.095 to -0.010) | 0.016 |  | 0.013 (-0.023 to 0.048) | 0.494 |  | - | - | - | - |
| **Orientation** |  |  |  |  |  |  |  |  |  |  |  |  |  |
| Normal | 0.001 (-0.005 to 0.007) | 0.710 |  | 0.005 (-0.002 to 0.012) | 0.193 |  | -0.004 (-0.023 to 0.014) | 0.671 |  | 0.002 (-0.002 to 0.007) | 0.322 | 0.0 | 0.597 |
| Borderline | Reference | / |  | Reference | / |  | Reference | / |  | Reference | / | / | / |
| High | -0.007 (-0.012 to -0.002) | 0.004 |  | -0.010 (-0.016 to -0.005) | <0.001 |  | -0.006 (-0.023 to 0.011) | 0.513 |  | -0.008 (-0.012 to -0.005) | <0.001 | 0.0 | 0.687 |

* After adjusting for sex, age, race (except for the CHARLS), body mass index, education, cohabitation status, current smoking, alcohol consumption, exercise, depressive symptoms, antihypertension medication, hypercholesterolemia, diabetes, coronary heart disease, stroke, cancer, and chronic lung disease.

^†^ The executive function scores were not pooled due to different tests.

**Table S9.** Comparison of baseline characteristics between participants included and excluded.

| **Characteristic** | **HRS** | | |  | **ELSA** | | |  | **CHARLS** | | |
| --- | --- | --- | --- | --- | --- | --- | --- | --- | --- | --- | --- |
|  | **Included**  **(n = 6964)** | **Excluded***  **(n = 7770)** | ***P* Value** |  | **Included**  **(n = 5334)** | **Excluded**  **(n = 6329)** | ***P* Value** |  | **Included**  **(n = 5292)** | **Excluded**  **(n = 6601)** | ***P* Value** |
| Women (%) | 4154 (59.6) | 4621 (59.5) | 0.827 |  | 2996 (56.2) | 3547 (56.0) | 0.893 |  | 2759 (52.1) | 3375 (51.1) | 0.275 |
| Age (years) | 66.3±8.0 | 73.4±9.9 | <0.001 |  | 62.4±8.9 | 65.4±12.3 | <0.001 |  | 58.1±8.8 | 58.3±10.1 | 0.114 |
| White (%) | 5745 (82.5) | 6345 (81.7) | 0.187 |  | 5237 (98.2) | 6092 (96.3) | <0.001 |  | 0 (0) | 0 (0) | - |
| Body mass index (kg/m2) | 29.5±5.6 | 28.6±6.1 | <0.001 |  | 27.5±4.5 | 27.6±4.8 | 0.362 |  | 23.7±3.7 | 23.7±3.8 | 0.735 |
| Systolic blood pressure (mmHg) | 130.4±19.3 | 135.5±22.5 | <0.001 |  | 140.7±19.1 | 143.9±21.6 | <0.001 |  | 129.6±20.7 | 131.7±22.1 | <0.001 |
| Diastolic blood pressure (mmHg) | 79.8±11.2 | 78.9±12.5 | 0.005 |  | 77.8±12.0 | 78.6±12.8 | 0.002 |  | 75.8±12.0 | 76.9±12.3 | <0.001 |
| High level of education (%) | 5710 (82.0) | 5486 (70.6) | <0.001 |  | 1875 (35.2) | 1513 (23.9) | <0.001 |  | 580 (11.0) | 1174 (17.8) | <0.001 |
| Living alone (%) | 1990 (28.6) | 3339 (43.0) | <0.001 |  | 1592 (29.8) | 2200 (34.8) | <0.001 |  | 532 (10.1) | 784 (11.9) | 0.002 |
| Current smoking (%) | 817 (11.7) | 993 (12.8) | 0.053 |  | 822 (15.4) | 1299 (20.5) | <0.001 |  | 2069 (39.1) | 2615 (39.6) | 0.565 |
| Current drinking (%) | 2567 (36.9) | 2166 (27.9) | <0.001 |  | 3335 (62.5) | 3489 (55.1) | <0.001 |  | 1353 (25.6) | 1701 (25.8) | 0.802 |
| Physically active (%) | 5560 (79.8) | 4955 (63.8) | <0.001 |  | 4424 (82.9) | 4363 (68.9) | <0.001 |  | 1629 (30.8) | 1662 (25.2) | <0.001 |
| Depressive symptoms (%) | 834 (12.0) | 1404 (18.1) | <0.001 |  | 707 (13.3) | 1189 (18.8) | <0.001 |  | 1258 (23.8) | 1530 (23.2) | 0.448 |
| History of diseases |  |  |  |  |  |  |  |  |  |  |  |
| Hypertension (%) | 4231 (60.8) | 5261 (67.7) | <0.001 |  | 1852 (34.7) | 2481 (39.2) | <0.001 |  | 2067 (39.1) | 2552 (38.7) | 0.658 |
| Diabetes (%) | 1193 (17.1) | 1818 (23.4) | <0.001 |  | 294 (5.5) | 519 (8.2) | <0.001 |  | 291 (5.5) | 403 (6.1) | 0.161 |
| Hypercholesterolemia (%) | 2999 (43.1) | 3511 (45.2) | 0.01 |  | 1961 (36.8) | 2059 (32.5) | <0.001 |  | 847 (16.0) | 1048 (15.9) | 0.849 |
| Coronary heart disease (%) | 1389 (19.9) | 2521 (32.4) | <0.001 |  | 874 (16.4) | 1343 (21.2) | <0.001 |  | 579 (10.9) | 869 (13.2) | <0.001 |
| Stroke (%) | 261 (3.7) | 748 (9.6) | <0.001 |  | 138 (2.6) | 312 (4.9) | <0.001 |  | 85 (1.6) | 130 (2.0) | 0.14 |
| Cancer (%) | 858 (12.3) | 1431 (18.4) | <0.001 |  | 294 (5.5) | 393 (6.2) | 0.111 |  | 45 (0.9) | 73 (1.1) | 0.162 |
| Chronic lung disease (%) | 518 (7.4) | 1013 (13.0) | <0.001 |  | 257 (4.8) | 485 (7.7) | <0.001 |  | 510 (9.6) | 672 (10.2) | 0.325 |
| Cognitive scores |  |  |  |  |  |  |  |  |  |  |  |
| Memory score | 10.6±3.2 | 8.7±3.7 | <0.001 |  | 10.3±3.2 | 8.8±3.7 | <0.001 |  | 15.1±4.8 | 15.0±5.0 | 0.427 |
| Executive score | 6 (5–7) | 6 (5–7) | <0.001 |  | 20.6±6.2 | 18.3±6.5 | <0.001 |  | 7 (4–8) | 6 (4–8) | 0.115 |
| Orientation score | 4 (4–4) | 4 (3–4) | <0.001 |  | 4 (4–4) | 4 (4–4) | <0.001 |  | 4 (3–4) | 4 (3–4) | 0.064 |

Participants excluded due to < 3 times of BP measurement or no follow-up cognitive score. Data are presented as mean ± SD, n (%), or median (IQR).

* *P* value for differences between included and excluded individuals.
